# Coordinated mechanisms of leaves and roots in response to drought stress underlying full-length transcriptome profiling in *Vicia sativa* L

**DOI:** 10.1186/s12870-020-02358-8

**Published:** 2020-04-15

**Authors:** Xueyang Min, Xiaoshan Lin, Boniface NDAYAMBAZA, Yanrong Wang, Wenxian Liu

**Affiliations:** State Key Laboratory of Grassland Agro-ecosystems, Lanzhou University; Key Laboratory of Grassland Livestock Industry Innovation, Ministry of Agriculture and Rural Affairs, China; Western China Technology Innovation Centre for Grassland Industry, Gansu Province, China; Engineering Research Center of Grassland Industry, Ministry of Education, China; College of Pastoral Agriculture Science and Technology, Lanzhou University, Lanzhou, 730000 People’s Republic of China

**Keywords:** Common vetch, Full-length transcripts, Drought stress, Leaves and roots

## Abstract

**Background:**

Common vetch (*Vicia sativa* L.) is an important self-pollinating annual forage legume and is of interest for drought prone regions as a protein source to feed livestock and human consumption. However, the development and production of common vetch are negatively affected by drought stress. Plants have evolved common or distinct metabolic pathways between the aboveground and underground in response to drought stress. Little is known regarding the coordinated response of aboveground and underground tissues of common vetch to drought stress.

**Results:**

Our results showed that a total of 30,427 full-length transcripts were identified in 12 samples, with an average length of 2278.89 bp. Global transcriptional profiles of the above 12 samples were then analysed via Illumina-Seq. A total of 3464 and 3062 differentially expressed genes (DEGs) were identified in the leaves and roots, respectively. Gene Ontology (GO) enrichment analyses identified that the dehydrin genes and Δ^1^-pyrroline-5-carboxylate synthase were induced for the biosynthesis of proline and water conservation. The Kyoto Encyclopedia of Genes and Genomes (KEGG) enrichment analysis results indicated that the DEGs were significantly enriched in hormone signal transduction, starch and sucrose metabolism, and arginine and proline metabolism, and various drought response candidate genes were also identified. Abscisic acid (ABA; the AREB/ABF-SnRK2 pathway) regulates the activity of AMY3 and BAM1 to induce starch degradation in leaves and increase carbon export to roots, which may be associated with the drought stress responses in common vetch. Among the co-induced transcription factors (TFs), AREB/ABF, bHLH, MYB, WRKY, and AP2/ERF had divergent expression patterns and may be key in the crosstalk between leaves and roots during adaption to drought stress. In transgenic yeast, the overexpression of four TFs increased yeast tolerance to osmotic stresses.

**Conclusion:**

The multipronged approach identified in the leaves and roots broadens our understanding of the coordinated mechanisms of drought response in common vetch, and further provides targets to improve drought resistance through genetic engineering.

## Background

Plants frequently encounter adverse growth conditions, such as drought, salinity, and extreme temperatures. These stresses can reduce plant growth and crop yield and play major roles in determining the geographic distribution of plant species [1–3]. Among these environmental factors, drought is one of the most acute abiotic stresses that adversely impacts plant productivity and survival [4, 5]. Under drought conditions, plants initiate a variety of complex signalling networks to adapt and survive during periods of water shortage, and three major sophisticated strategies have evolved to respond to drought conditions: (i) stress escape, (ii) stress avoidance and (iii) stress tolerance [6]. Drought escape occurs during a severely drought-shortened growing season and through the response to environmental cues that change plant molecular mechanisms. Conversely, drought avoidance occurs through a series of morphological and physiological changes that increase plant water-use efficiency and decrease transpiration. Drought tolerance occurs through the production of molecules that stabilize proteins and osmotic adjustments to withstand dehydration [7, 8]. These plant adaption strategies include morphological, biochemical, physiological and molecular changes underpinned by alterations in the expression of numerous genes that may include the upregulation of stress signal transduction-related genes, functional proteins and transcription factors (TFs) [9, 10].

The important genes participating in drought resistance are generally classified into two groups: regulatory genes and functional genes [2, 10]. Functional genes encode important metabolic proteins and enzymes that directly function in the protection of cells against stress, and the regulatory genes encode numerous regulatory proteins, including protein phosphatases, kinases, and TFs, that mainly play important roles in synchronizing gene expression and signal transduction in abiotic stress responses [11, 12]. It is especially noteworthy that up to 10% of genes in plant genome are TFs, which could be activated through abscisic acid (ABA) dependent/independent pathways and play particularly pivotal roles in drought tolerance in plants by regulating the downstream stress-responsive genes [5, 13].

The coordination of metabolic assimilation is a key factors for plants’ adaptive mechanisms to drought stress. In plants, most of the water transpires from the soil in exchange for CO_2_, making water availability a major limiting factor for growth and productivity. Although the aboveground leaves and underground roots have distinct developmental trajectories, plants also evolved highly coordinated biological processes to acclimate to drought conditions by fine-tuning energy production in leaves in response to the availability of water and nutrients in roots [3, 14]. Considering the complexity of the biological processes between above- and underground tissues, it is essential to understand the common and specific stress-related genes expression profiles and molecular networks involved in drought resistance on a whole-transcriptome level in plants. Using next-generation RNA sequencing (NGS) technology, numerous drought-responsive genes have been identified in many non-model plant species including *Pinus halepensis* [15], *Chenopodium quinoa* (Willd.) [16], and *Ammopiptanthus mongolicus* [17], but these studies mainly focused on single tissues or whole plants, except for *Prunus mahaleb*, *Arundo donax*, *Lens culinaris*, and *Prunus persica,* for which NGS was used to recognize the response mechanisms coordinated between leaves and roots under drought stress [18–22]. These studies indicated that hormone signal transduction plays a considerable role in the plant response to drought stress. Compare to the NGS method, the recently developed Pacific Biosciences (PacBio) full-length sequencing can produce longer reads and highly accurate and unbiased sequences, making it much more effective for unsolved problems in genome, transcriptome, and epigenetics research in a species without a reference genome [23, 24]. However, to our knowledge, genome-wide transcriptomic study of drought-responsive genes in plants with this new full-length sequencing approach has not yet been reported.

Common vetch (*Vicia sativa* L.) is an important self-pollinating annual forage legume and is of interest for drought prone regions as a good quality animal feedstock with minimal input [25, 26]. Owing to its low cost, high nutritional value and broad environmental adaptation, common vetch not only has been used as a protein source to feed livestock but also for human consumption [27, 28]. When compared to *V. narbonensis* and *V. villosa*, the growth of common vetch was most affected under water-limited conditions [29]. Therefore, systematically recognising the molecular mechanisms of the response to drought is essential for the improvement of the quality and ecological distribution of common vetch. Previously, Zhu et al. (2019) performed a de novo transcriptional analysis of whole common vetch plants under drought stress and revealed that drought-responsive genes are mainly involved in plant hormone signal transduction, glycolysis/gluconeogenesis and phenylpropanoid biosynthesis [30]. However, given that most of the sequencing results from NGS cannot represent full-length cDNA sequences, and the authors only focused on how whole common vetch plants response to drought stress, additional efforts are needed to elucidate and compare the molecular mechanisms systematically between above- and underground tissues of common vetch during the response to drought with full-length sequencing approach. In the present study, for the firsr time we generated the full-length transcriptome of common vetch with a PacBio full-length sequencing approach. The differentially expressed genes and the common and different molecular mechanisms of leaves and roots in response to drought stress were further systematically identified and analysed. These results would deep our understanding of the coordinated molecular mechanisms involved in the response to drought stress and meanwhile accelerate drought tolerance related gene discovery and genetic improvement in common vetch.

## Results

### Illumina-Seq and mapping

**Fig. 1:**
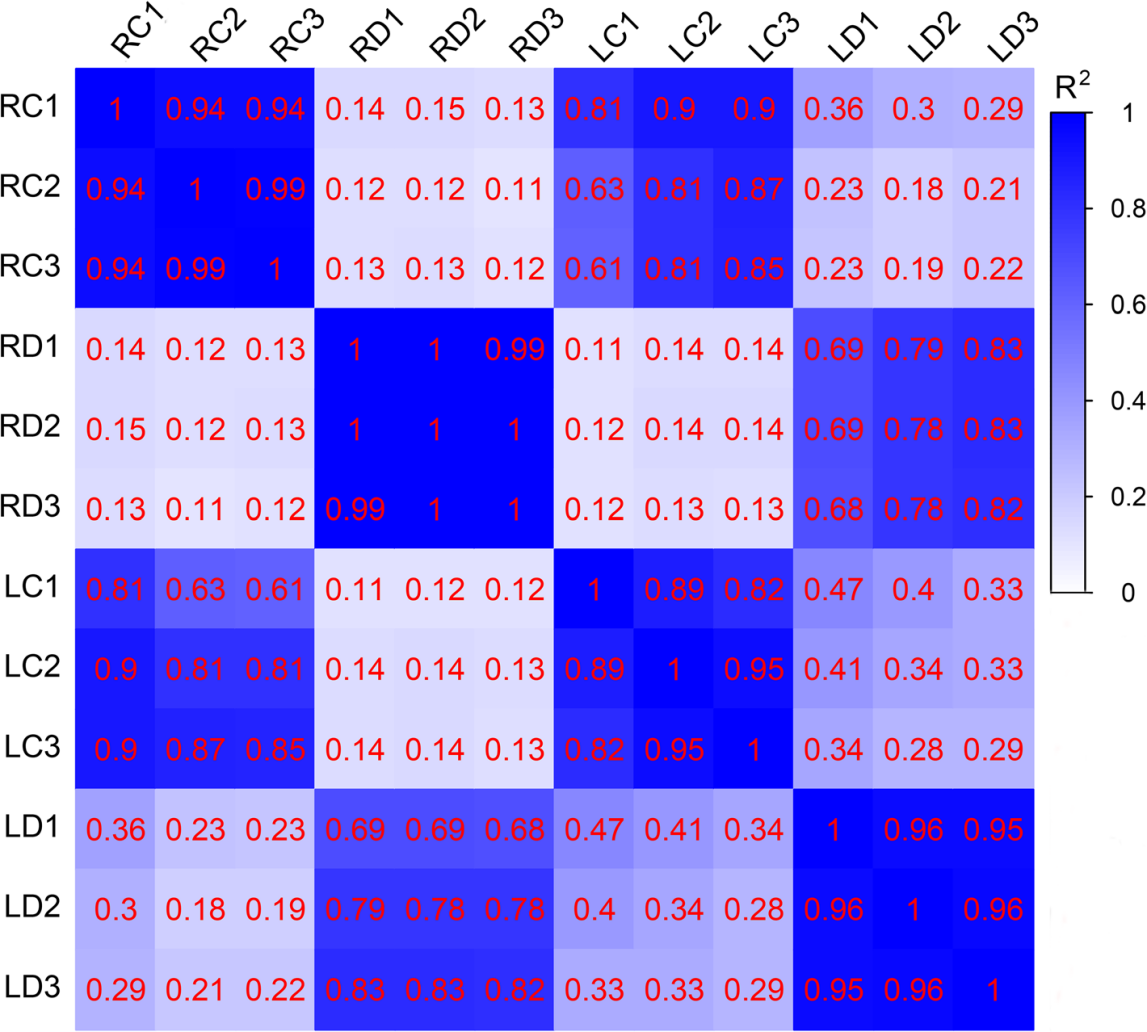
Pearson correlation between twelve samples. RC represents the root control groups; LC represents the leaf control groups; RD represents the PEG-treated root groups; LD represents the PEG-treated leaf groups. *R*^*2*^ represents the correlation coefficient. The blue background represents a greater correlation coefficient

As aboveground leaves are the first tissue to sense water loss and underground roots are the first organs to be exposed to drought, they follow distinct biological processes in response to drought stress. In this study, both leaf and root tissues were collected from the control and drought conditions and used for transcriptome analysis to obtain an overview of the responses of common vetch during water deprivation. In our preliminary experiment, we found that high concentrations of PEG caused severe inhibition of common vetch seedling growth, and thus, moderate concentrations (20% PEG) were used for transcriptome analyses in this study (Additional file 8: Fig. S1). Twelve cDNA libraries from the roots and leaves that had been exposed to water deficit for 24 h (RD1, RD2, RD3, LD1, LD2, and LD3) and roots and leaves under control conditions (RC1, RC2, RC3, LC1, LC2 and LC3) were prepared for Illumina sequencing. In total, 318.77 million paired-end reads were generated after filtering out low-quality reads that had a Q30 percentage greater than 90.35%. Among them, 160.27 and 158.5 million clean reads were obtained from the roots and leaves, respectively. The average GC content of clean reads was 43.07% (Table 1). The Pearson’s correlation coefficient showed that all correlation values between the 3 replicates ranged from 0.82 to 1, indicating a perfect positive correlation and that the sequencing results could be used for further studies (Fig. 1).

**Table 1:** Summary of RNA sequencing data in million (M) reads from 12 RNA libraries and comparison with full length transcripts

Libraries	Clean data				Mapped reads (Ratio)			
	Pairend Reads (M)	Base Sum (M)	GC (%)	Q30 (%)	Total Reads (M)	Mapped Reads (%)	Uniq mapped Reads (%)	Multi mapped Reads (%)
RC1	26.92	804.55	42.93	90.68	26.92	76.02%	30.23%	69.77%
RC2	26.57	793.00	42.69	90.77	26.57	76.75%	30.79%	69.21%
RC3	25.31	755.13	42.79	90.35	25.31	76.09%	30.89%	69.11%
RD1	27.76	8301.00	43.66	92.19	25.65	77.30%	28.59%	71.41%
RD2	27.33	8154.65	43.38	92.15	27.02	75.21%	31.17%	68.83%
RD3	26.38	7873.64	42.69	92.42	28.01	74.63%	30.23%	69.77%
LC1	25.65	7672.36	42.65	92.32	27.76	79.42%	26.51%	73.49%
LC2	27.02	8079.60	42.69	92.62	27.33	79.72%	26.62%	73.38%
LC3	28.01	8374.75	42.77	91.78	26.38	79.45%	26.01%	73.99%
LD1	25.13	7496.12	43.6	92.11	25.13	79.69%	24.45%	75.55%
LD2	25.75	7686.24	43.96	91.95	25.75	78.87%	23.52%	76.48%
LD3	26.94	8042.36	43.05	92.11	26.94	77.65%	25.62%	74.38%
Mean	26.56	6169.45	43.07	91.79	26.56	77.57%	27.89%	72.11%

**Fig. 2:**
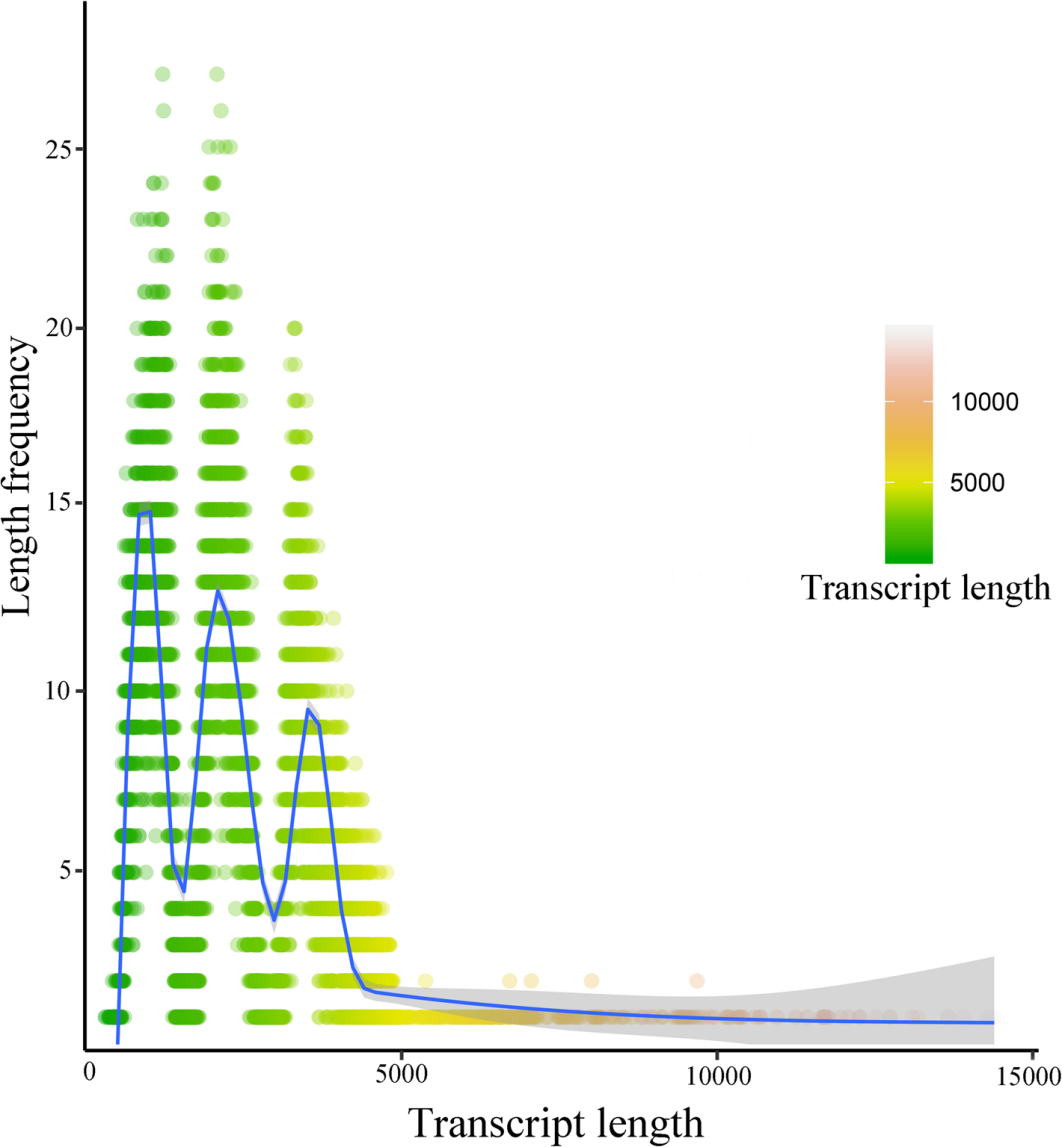
Length distribution of the full transcripts in common vetch

Equal RNAs extracted from 12 samples were pooled as one sample and subjected to SMRT sequencing to generate an informative reference transcriptome database. These were combined with NGS sequences to improve the quality and number of correct subreads. Finally, a total of 30,427 transcripts were identified in all 12 samples. The clean reads were then mapped onto the full-length transcripts via Bowtie2 software. Overall, the average, unique, and multi-mapped ratios for each library were 77.57, 27.89, and 72.11%, respectively (Table 1). The lengths of these 30,427 transcripts ranged from 304 to 14,390 bp, and the average length was 2278.89 bp, with an N50 length of 4604 bp (Fig. 2). The average length of novel transcripts was found to be shorter (1682.75 bp) than that of annotated transcripts.

### Transcript annotation and classification in public databases

**Fig. 3:**
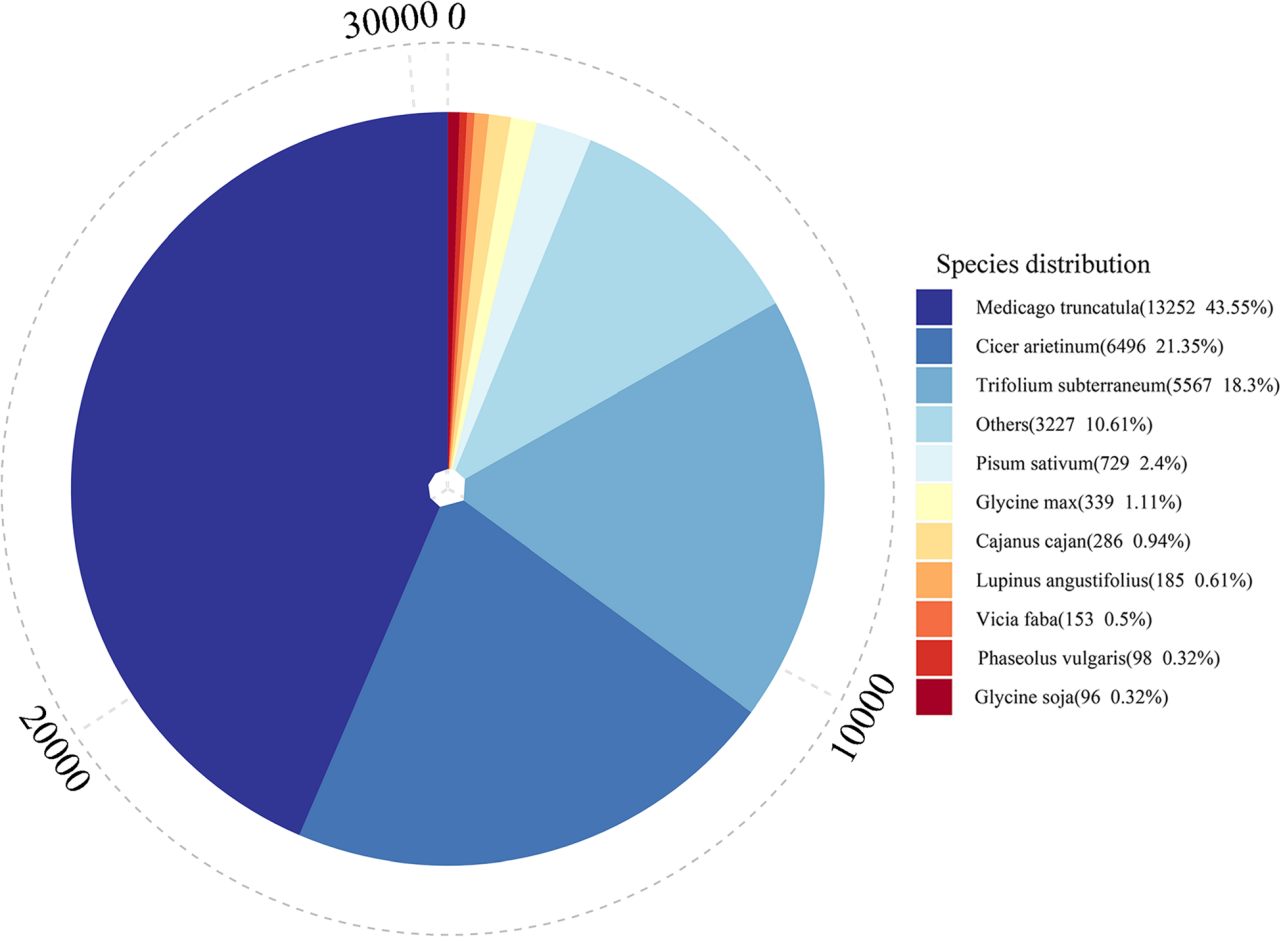
The Top-Hit species distribution of transcripts that were annotated on the basis of homology with genes from closely related species

To predict the functional annotation of the transcripts, all assembled transcript sequences were aligned against eight public databases, including the Nr, KOG, COG, Pfam, Swiss-Prot, eggNOG, GO and KEGG databases. The results are shown in Table 2**.** A total of 29,898 transcripts were successfully annotated in these 8 databases. The number of annotated transcripts ranged from 10,220 (33.59%, COG) to 29,824 (98.01%, Nr), and 8107 (26.64%) and 528 (1.74%) transcripts were annotated in all databases and in no database, respectively. Compared with other species, *Medicago truncatula* showed the most matches to common vetch (13,252, 43.55%), followed by *Cicer arietinum* (6496, 21.35%) and *Trifolium subterraneum* (5567, 18.30%) (Fig. 3).

**Table 2:** Success rate statistics of transcripts annotation

Term	Number of transcripts	Percentage
Annotated in COG	10,220	33.59%
Annotated in GO	19,631	64.52%
Annotated in KEGG	13,232	43.49%
Annotated in KOG	19,012	62.48%
Annotated in PFAM	25,229	82.91%
Annotated in Swiss Prot	23,441	77.04%
Annotated in eggNOG	28,532	93.77%
Annotated in NR	29,824	98.01%
Annotated in all databases	8107	26.64%
No database annotated	529	1.74%

### Analysis of potential DEGs in leaves and roots

**Fig. 4:**
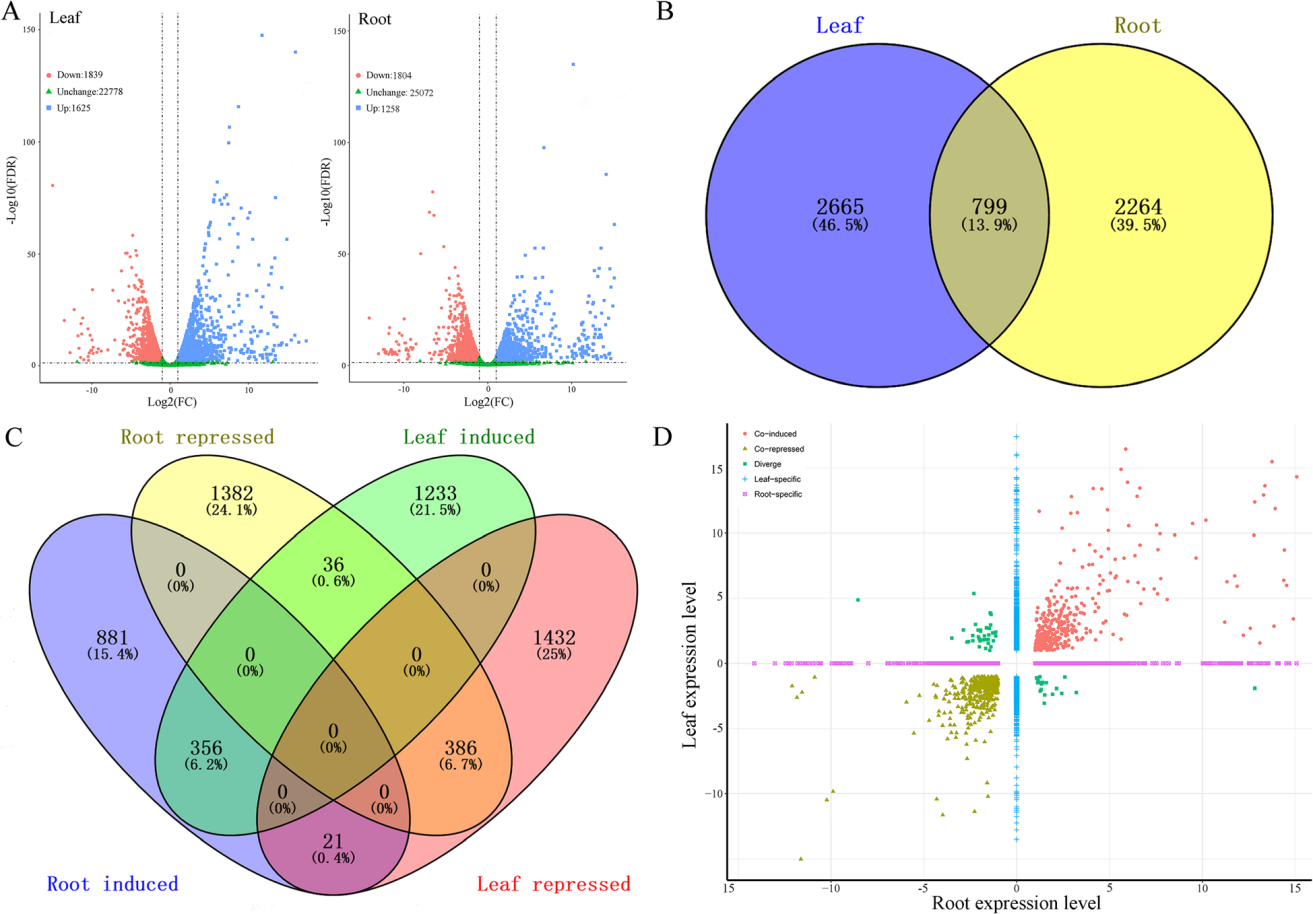
**a** volcano plot of DEGs in leaves and roots under drought stress. Red and blue dots represent the downregulated and upregulated transcripts, respectively. **b** Venn diagram represents the number of overlapping DEGs between leaves and roots. **c** Comparison of drought-induced DEGs across leaf and root tissues. **d** Scatter plot indicates the gene expression levels that were co-induced, co-repressed, diverged and specifically regulated by drought stress

To further gain insight into the transcriptomic profiles of the response of common vetch to drought stress, genes in the leaf and root tissues were compared between the control and drought stress conditions. A total of 3464 and 3062 DEGs were identified in the leaves and roots, respectively, under drought conditions compared with control conditions. Among these DEGs, 1625 and 1258 genes were upregulated in leaves and roots, respectively, while 1839 and 1804 DEGs were downregulated in leaves and roots under drought stress, respectively (Fig. 4a). These results suggest that leaves are more sensitive to drought stress than roots. Among them, 2665 (46.5%) and 2264 (39.5%) DEGs were found to be leaf and root-specific, respectively (Fig. 4b). Notably, a total of 799 (13.9%) genes were differentially expressed in both tissues; of the 799 genes, 356 (6.2%) were co-induced, 386 (6.7%) were co-repressed and 57 (1%) were oppositely expressed in the leaves and roots (Fig. 4c and d). The absolute value of the DEGs indicated that there were fewer highly expressed genes among co-repressed DEGs than among co-induced DEGs under drought conditions in both tissues (Fig. 4d). The gene expression patterns of 528 novel transcripts in response to drought stress in common vetch were also analysed. The analysis revealed a total of 40 and 30 significantly differentially expressed transcripts in the leaves and roots under drought stress, respectively. In the leaves, 26 transcripts were upregulated, 14 were downregulated, 392 were normally expressed, and 96 were not detected. In the roots, 16 transcripts were upregulated, 14 were downregulated, 441 were normally expressed, and 57 were not detected (Additional file 1: Table S1). For the average expression changes, all detected transcripts in both tissues showed a very similar level, but more highly expressed transcripts were found in leaves than in roots under drought conditions (Additional file 9: Fig. S2A). For the novel transcripts, their expression patterns are more likely to be upregulated in both tissues (Additional file 9: Fig. S2B).

**Fig. 5:**
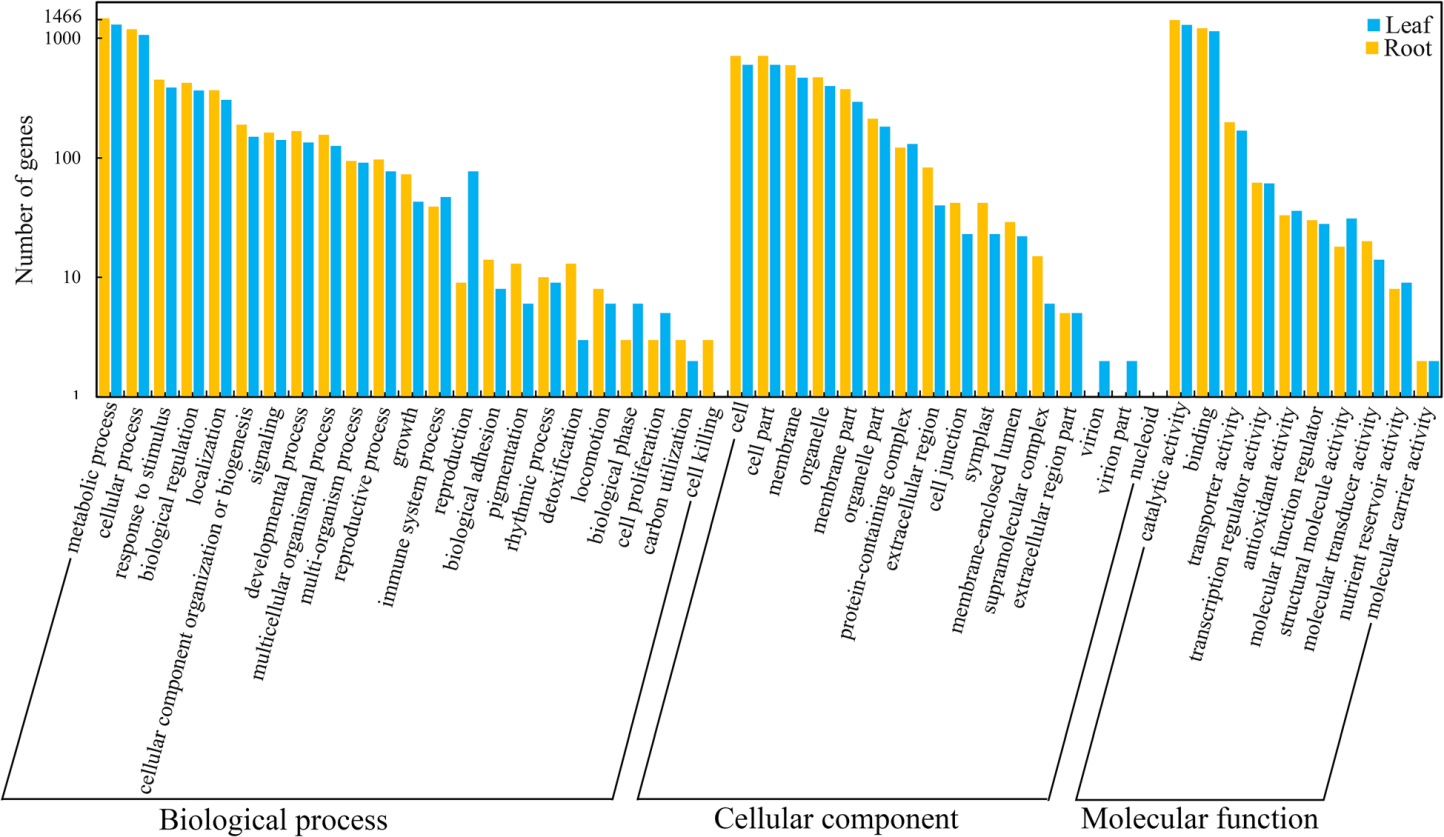
Histogram of GO terms assigned to DEGs in leaves and roots. The DEGs are categorized into three main groups: cellular components (CCs), molecular functions (MFs), and biological processes (BPs)

To further understand whether similar mechanisms occurred in the leaves and roots of common vetch in response to drought stress, the GO database was used to classify DEG functions. In total, 49 functional groups were distributed into three categories. Under the biological process (BP) category, metabolic process was the largest group in the leaves (1594: 42.8% upregulated) and roots (1423: 41.7% upregulated), followed by cellular process in the leaves (1273: 41.7% upregulated) and roots (1151: 42.5% upregulated), single-organism process in the leaves (1074: 45.2% upregulated) and roots (943: 42.5% and upregulated), and response to stimulus in the leaves (446: 50.2% upregulated) and roots (385: 40.5% upregulated). There were more upregulated DEGs involved in “response to stimulus” in the leaves than in the roots, suggesting that these DEGs may play crucial functions in drought sensing and response. In the cellular component (CC) category, the DEGs associated with “cell” in the leaves (714: 54.6% upregulated) and roots (598: 53.8% upregulated), “cell part” in the leaves (714: 50.2% upregulated) and roots (599: 53.9% upregulated), and “membrane” in the leaves (596: 32.4% upregulated) and roots (469: 41.2% upregulated) represented the most abundant categories. Among the top three GO terms in the molecular function (MF) group, “catalytic activity” in the leaves (1410: 41.4% upregulated) and roots (1286: 40.0% upregulated), “binding” in the leaves (1207: 45.4% upregulated) and roots (1142: 38.3% upregulated) and “transporter activity” in the leaves (200: 37.5% upregulated) and roots (173: 37.6% upregulated) were more enriched than other terms (Fig. 5). Of the 3464 DEGs in the leaves, 2293 (66.2%) were successfully annotated in the GO database; however, 69 DEGs did not match any genes in the eight public databases, and only one transcript was detected in both tissues. The remaining DEGs were tissue-specific, which may suggest the presence of novel transcripts that have tissue-specific functions.

### Verification of gene expression

To further confirm the reliability of our RNA-Seq data, 10 candidate DEGs were randomly selected from the leaves and roots for qRT-PCR validation. As shown in additional file 10: Fig. S3, the expression levels of these DEGs in both leaves and roots significantly correlated with the FPKM values. Among them, five genes were upregulated, three were downregulated, and two were oppositely expressed after drought treatment. In our study, the linear regression analysis showed a high positive correlation coefficient (*R*^*2*^ = 0.81) between the values of the qRT-PCR and RNA-Seq methods, indicating that the expression of these DEGs in our transcript data generally agreed with the qRT-PCR results (Additional file 11: Fig. S4).

### GO enrichment analysis

**Fig. 6:**
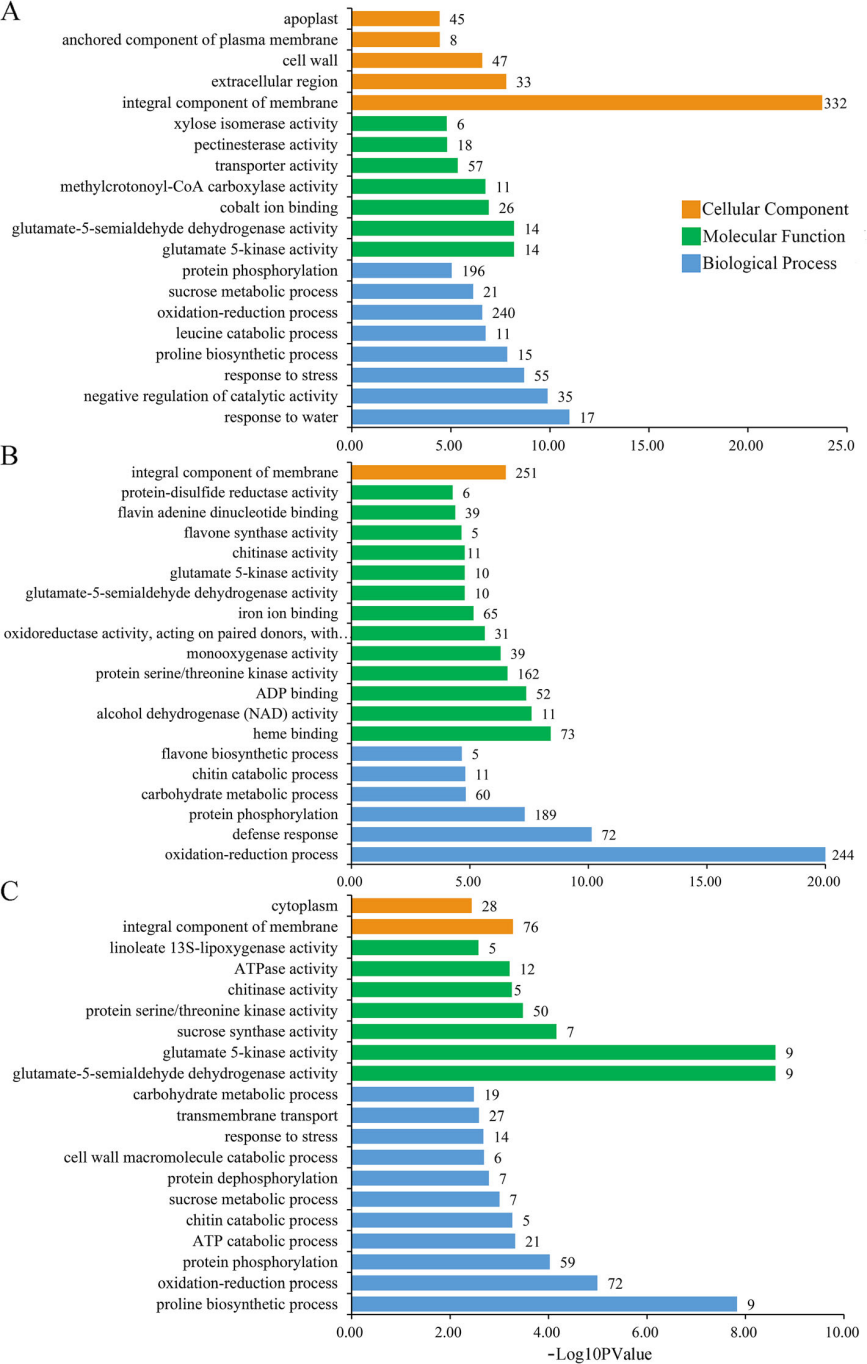
GO enrichment analysis of the top 20 most strongly represented categories. The DEGs obtained from leaves (**a**), roots (**b**) and the DEGs shared between two tissues (**c**) were assigned into three main categories: CCs, MFs, and BPs. The names of the GO categories are listed along the y-axis. The degree of GO enrichment is represented by the -Log_10_PValue and the number of transcripts enriched in each category

Plant leaves and roots follow distinct developmental trajectories, and to adapt to fluctuating environments, their biological processes have become highly coordinated at the whole-plant level. To investigate the molecular mechanisms that govern the interaction and coordination of whole plants under drought stress, we performed GO enrichment (top 20) analysis of the leaves, roots, and DEGs shared between the two tissues. GO enrichment showed that drought stress significantly affected eight BP, seven MF, and five CC categories in the leaves of common vetch (Fig. 6a). Similarly, thirteen MF categories, six BP categories, and one CC metabolic category were affected in the roots of common vetch (Fig. 6b). The terms from the MF category in the roots were enriched with more DEGs than those from the leaves. Furthermore, eleven BPs, seven MFs, and two CC categories were significantly enriched in both tissues (Fig. 6c). The “integral component of membrane” was the most significantly enriched category in leaves, followed by “negative regulation of catalytic activity” and “response to water”. The expression and accumulation of plant dehydrin genes were consistently positively correlated with resistance to abiotic stress. Notably, all 17 genes enriched in the “response to water” category were upregulated and annotated as “dehydrin” in the Nr database (Additional file 12: Fig. S5A). Among them, 14 were significantly upregulated (Log_2_FC > 6), while their expression was barely detected under control conditions. The DEGs most significantly enriched in the roots were “oxidation-reduction process”, followed by “defence response” and “protein phosphorylation” in the BP category (Additional file 12: Fig.S5B). Among the 244 enriched DEGs in the “oxidation-reduction process” category, 172 genes were specifically expressed in the roots, with 68 upregulated and 104 downregulated (Additional file 2: Table S2). Notably, 72 DEGs were detected in both tissues, among which 30 were upregulated, 36 were downregulated and six were oppositely expressed in the leaves and roots. The oxidoreductase activity was also enriched in the main MF category. The results implied that DEGs related to oxidation/reduction activity might play an important role in the response to drought stress. “Defence response” was the second most enriched category among the top 20 GO terms in the roots. Interestingly, 18 DEGs were detected in both tissues, among which one was upregulated, eight were downregulated, and nine were oppositely expressed in the leaves and roots, indicating that these oppositely expressed genes may play different roles in response to drought stress in above- and underground tissues of common vetch (Additional file 3: Table S3). Further, there were 799 DEGs in both tissues we analysed, and among the top 20 GO terms, the “glutamate-5-semialdehyde dehydrogenase activity” term was the most significantly enriched, followed by “glutamate 5-kinase activity”, “proline biosynthetic process”, and “oxidation-reduction process” (Additional file 12: Fig. S5C). Among 14 DEGs enriched in the “response to stress” pathway, 10 were upregulated and 3 were downregulated in both tissues, and one was oppositely expressed, which was upregulated in the leaves and downregulated in the roots. The “proline biosynthetic process” term was the most enriched term in the BP category. In total, nine Δ^1^-pyrroline-5-carboxylate synthases (P5CSs) were enriched in this term, and all of them were upregulated.

### KEGG enrichment analysis

**Fig. 7:**
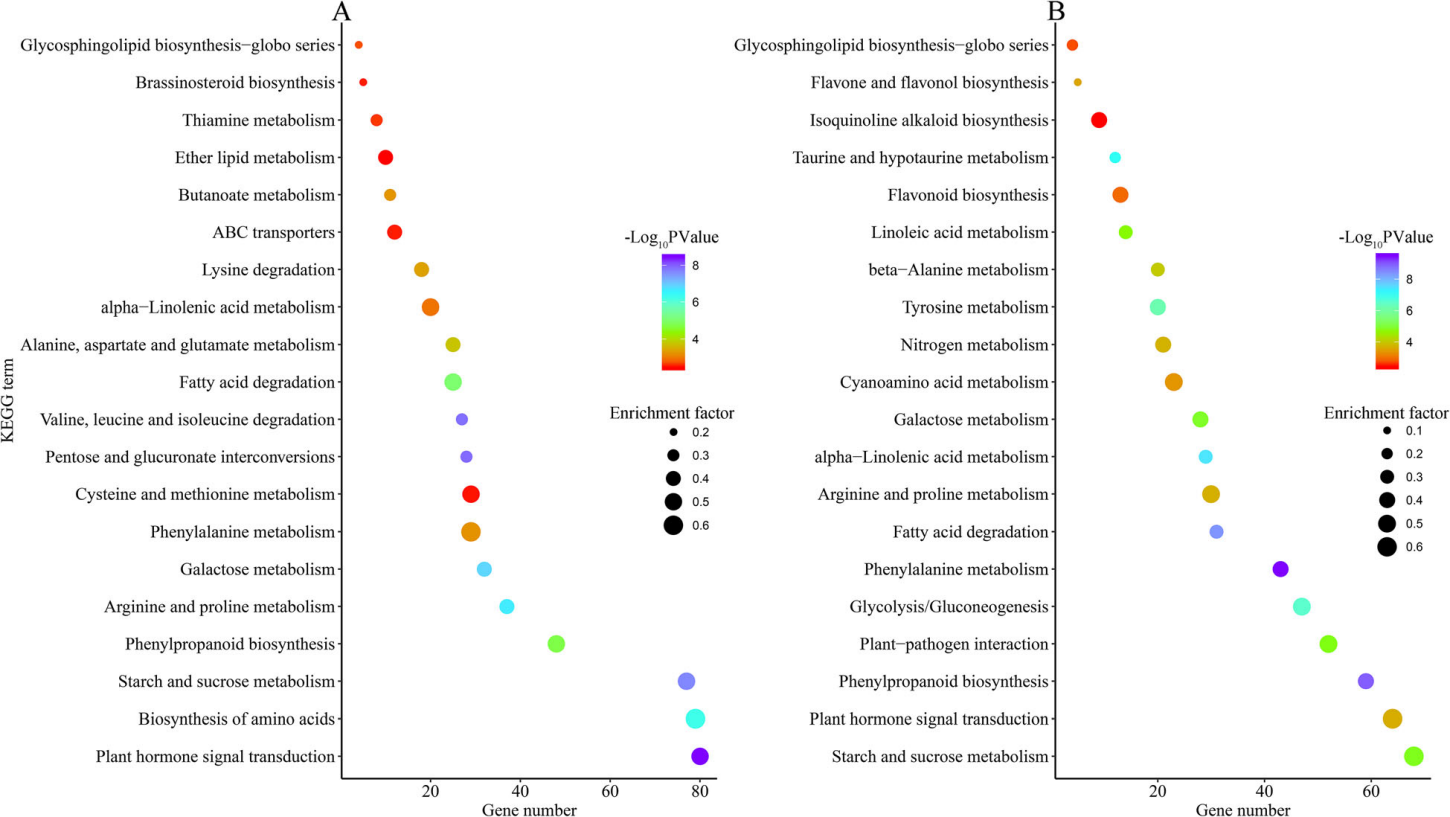
Scatterplot of enriched KEGG pathways for DEGs under drought stress. Only the top 20 most strongly represented pathways are displayed. The enrichment factor is the ratio of the total of annotated genes to the DEG number in a certain pathway. The colour of the dots represents the range of the -log_10_ (*P*-Value)

To investigate the complex biological behaviours of the DEGs involved in drought stress, KEGG pathway enrichment analyses were carried out. A total of 1424 drought-responsive DEGs were assigned to 113 KEGG pathways in the leaves and roots (Additional file 4: Table S4). The top 20 potential pathways of the leaves and roots were screened as the most intensive response activities (Fig. 7). From the KEGG enrichment analysis, “plant hormone signal transduction”, “biosynthesis of amino acids”, “starch and sucrose metabolism”, “phenylpropanoid biosynthesis”, and “arginine and proline metabolism” were the most active physiological activities in the leaves, while “plant hormone signal transduction”, “starch and sucrose metabolism”, “phenylpropanoid biosynthesis”, “plant-pathogen interaction”, “glycolysis/gluconeogenesis”, and “phenylalanine metabolism” were the most active activities in the roots. “Plant hormone signal transduction”, “starch and sucrose metabolism” and “phenylpropanoid biosynthesis” were the most enriched pathways in both tissues responding to drought. There were nine common and eleven different pathways in both tissues, and more than half of them were classified as “metabolism”-related pathways.

**Fig. 8:**
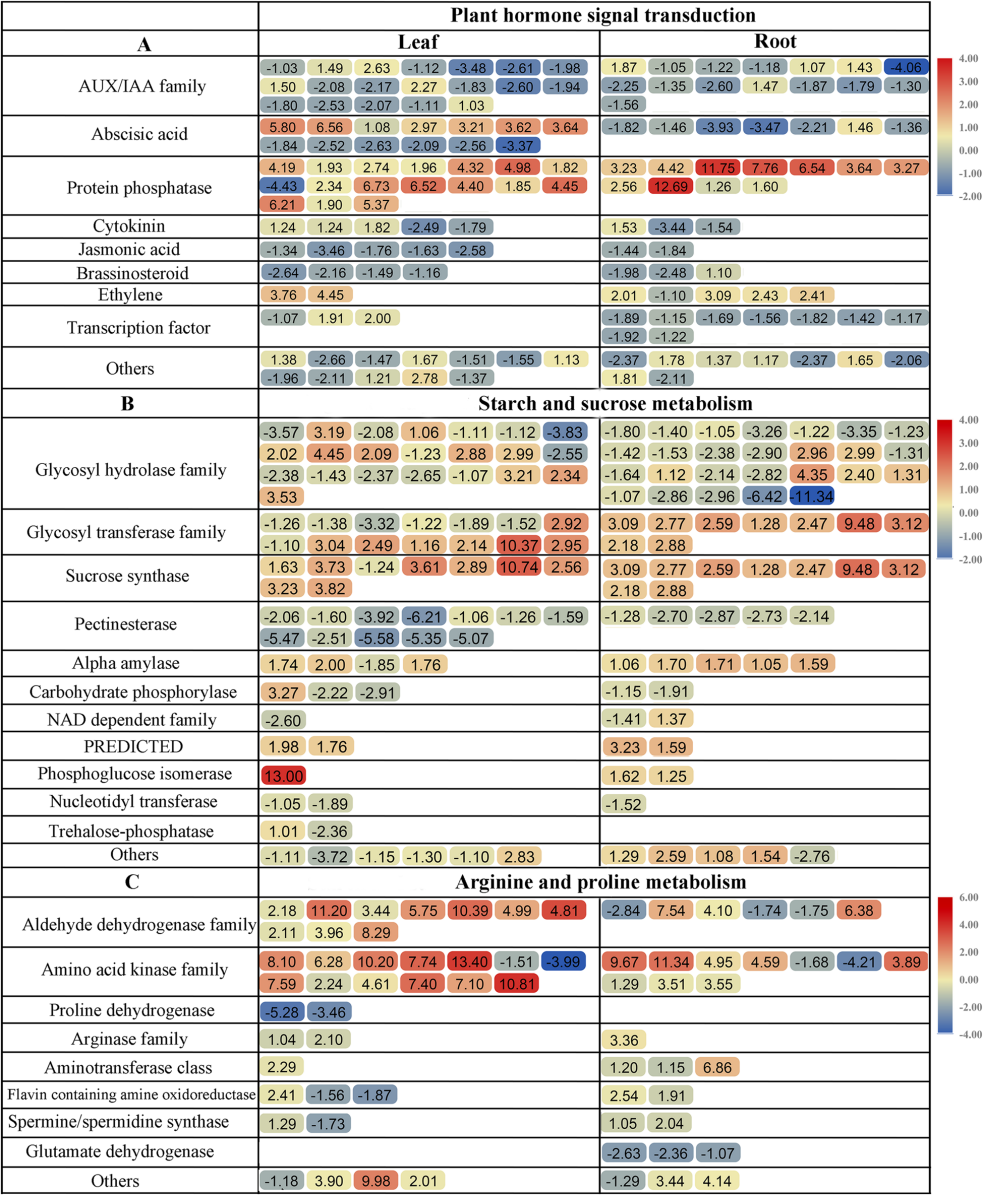
The expression level of DEGs identified in leaves and roots. **a**, **b** and **d** displayed the DEGs involved in “Plant hormone signal transduction”, “Starch and sucrose metabolism” and “Arginine and proline metabolism” in common vetch, respectively. The scale bar on the right represents the observed changes in expression in terms of Log_2_FC from upregulation (red) to downregulation (blue)

We further compared three enriched KEGG pathways between the leaves and roots: the “plant hormone signal transduction” (ko04075), “arginine and proline metabolism” (ko00330), and “starch and sucrose metabolism” (ko00500) pathways. The identification of several hormone-related genes indicated that common vetch may use a large array of signalling mediators to combat drought stress. After drought treatment, several plant hormone signal-related DEGs were up- or downregulated in the leaves and roots, such as the ABA, auxin, phosphatase, cytokinin (CK), brassinosteroid (BR), ethylene (ET), gibberellin (GA), and jasmonic acid (JA) signalling pathways. As shown in Fig. 8a and additional file 5: Table S5a, in the plant hormone signal transduction pathway, most genes in the leaves or roots were involved in the ABA, auxin, and phosphatase pathways; all phosphatase-related genes were upregulated in the roots, while most of them were downregulated in the leaves. Most of the ABA metabolism-related genes were downregulated in both tissues. Interestingly, the TFs participating in plant hormone metabolism were identified only in the roots, and all of them were downregulated. As shown in Fig. 8b and additional file 5: Table S5b, in the plant “starch and sucrose metabolism” pathway, most genes in the leaves or roots were involved in the glycosyl hydrolase family; nearly half of these genes were upregulated in the leaves, while 80.76% of genes were downregulated in the roots. In contrast, glycosyl transferase family genes were all upregulated in the roots, and half of them were upregulated in the leaves. Sucrose synthase genes were specifically enriched in leaves, and most of them were upregulated. In this study, the “arginine and proline metabolism” pathway was the most redundant among the genes, and a large number of them exhibited upregulation under drought conditions (Fig. 8c and additional file 5: Table S5c). A number of amino acid kinase family and aldehyde dehydrogenase (ALDH) family genes were prominently expressed in both leaves and roots. There were more upregulated DEGs enriched in the leaves than in the roots. All ALDHs were upregulated in the leaves, and their transcript abundance in the leaves was much greater than that in the roots. In contrast, proline dehydrogenase (*PDH*) and spermine/spermidine synthase-related genes were specifically expressed in the leaves and roots, respectively.

### Identification of TFs in response to drought stress

**Fig. 9:**
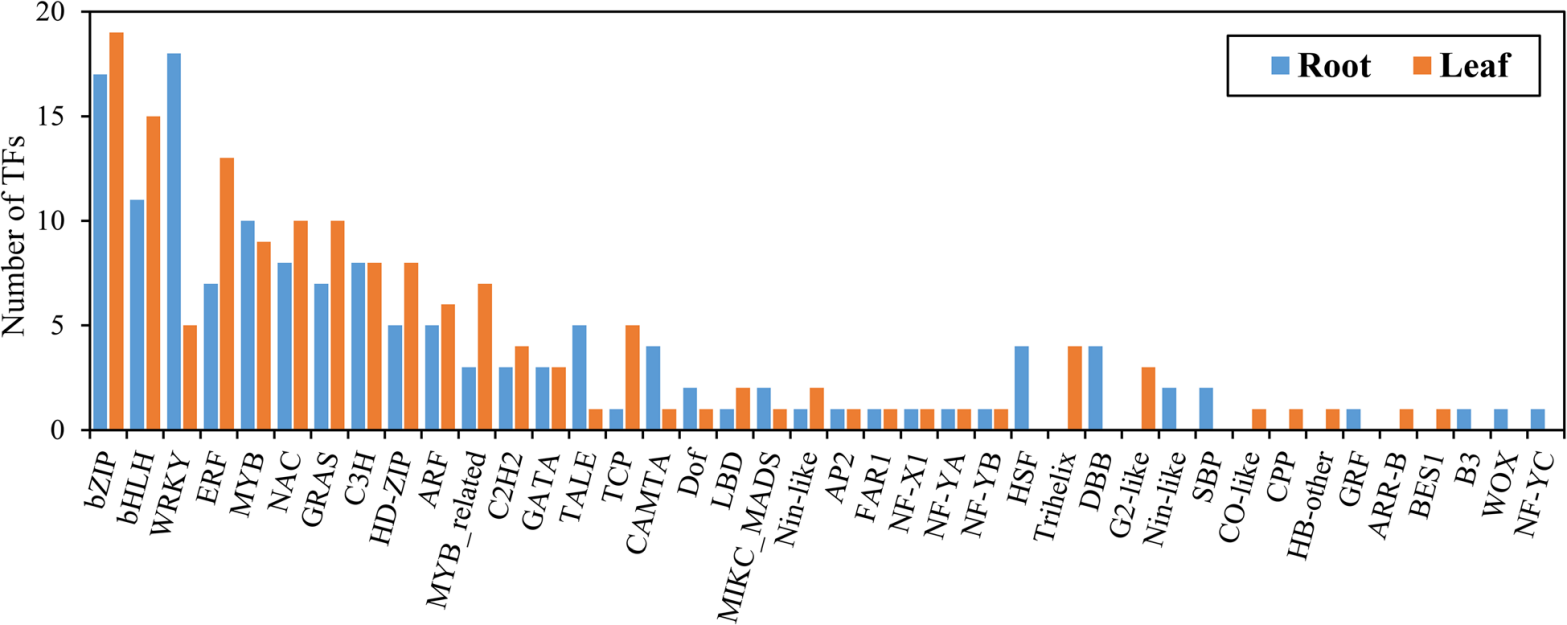
Distribution of transcription factors responsive to drought stress in common vetch. Data are sorted by the number of DEGs in both leaves and roots. Blue bar represent root; orange bar represent leaf

**Fig. 10:**
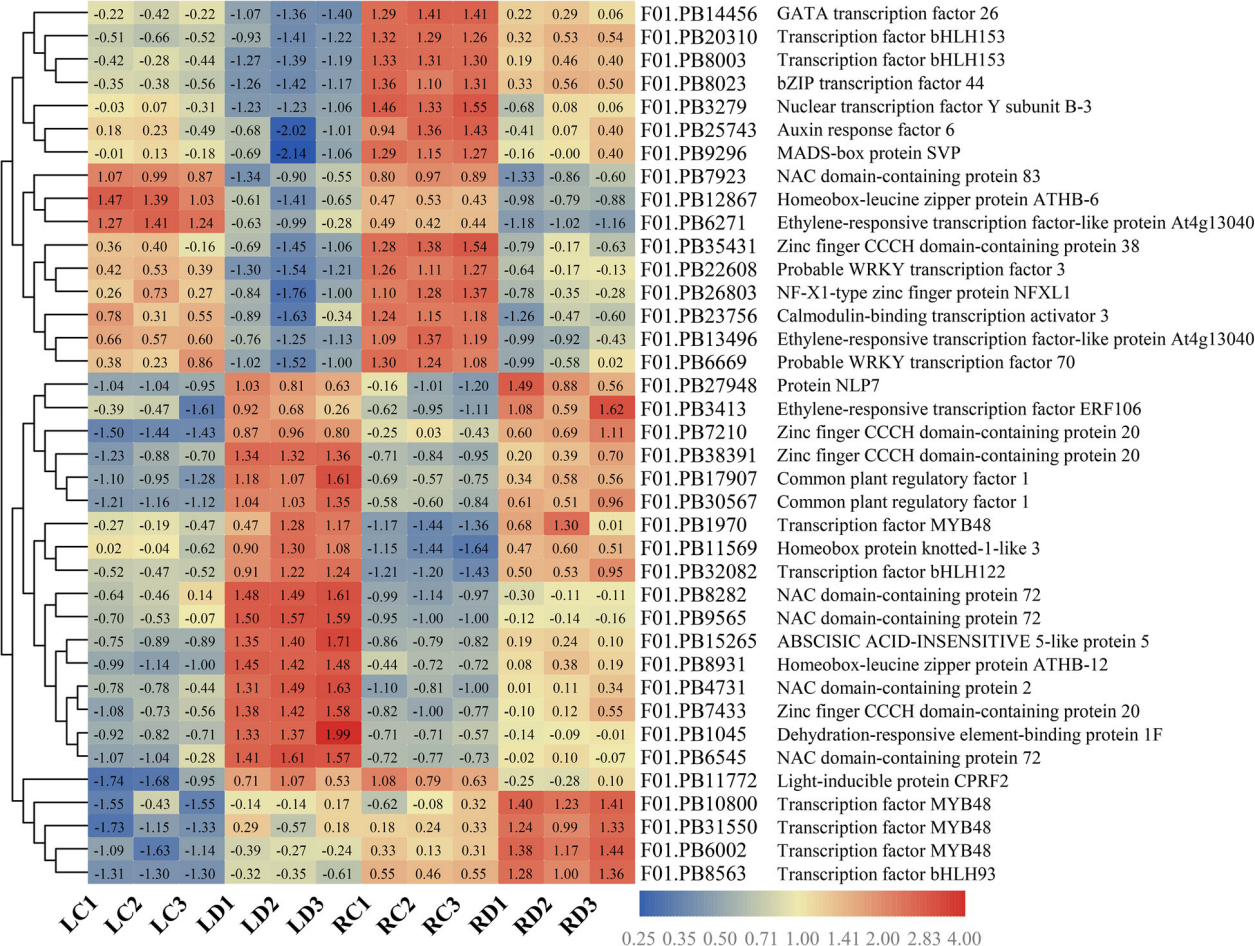
Heat map of the 38 co-responsive transcription factor DEGs in leaves and roots. The colours indicate the abundance of transcripts calculated as Log_2_ (FPKM) in the control and drought-stressed plants (see color key). Further information about each gene is provided in the right

TFs play crucial roles in modulating the stress response during plant survival under severe environmental conditions. In our transcriptome data, a total of 149 and 133 DEGs were identified as TFs and classified into 32 and 33 families in the leaves and roots, respectively (Fig. 9). The most represented TF families in common vetch leaves and roots were bZIP, bHLH, WRKY, ERF, MYB, and NAC, which are known as stress-related TFs and have been well studied for their roles in mediating drought stress responses in plants. The number of WRKY transcripts in the leaves was nearly four times that in the roots, while the number of ERFs identified in the leaves was nearly two times that in the roots. In total, 38 co-responsive DEGs were identified as TFs, which belonged to 17 families (Fig. 10). Among these TFs, the most abundant TF families were bHLH (5) and NAC (5), followed by the bHLH (4), ERF (4), and C3H (4) families. There were 111 and 95 TFs identified as specifically responsive in the leaves and roots, respectively; for example, HSF, Trihelix, G2-like, CO-like, CPP, ARR-B and BES1 were specifically expressed in leaves, indicating tissue-specific mechanisms of drought stress tolerance in common vetch.

### Functional verification of candidate TFs in yeast

**Fig. 11:**
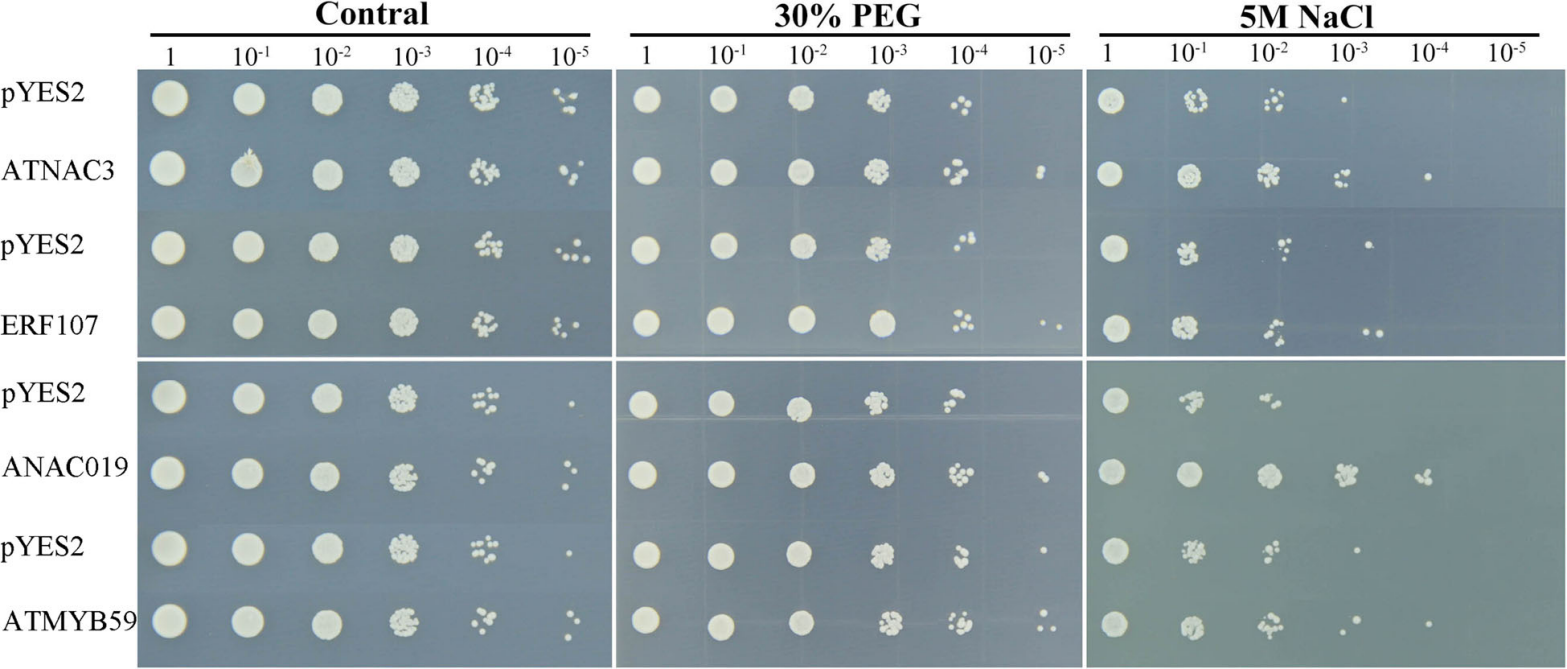
Phenotypic growth assays of *Saccharomyces cerevisiae* INVSc1 cells transformed with the pYES2 empty vector, pYES2-ATNAC3, pYES2-ERF107, pYES2-ANAC019 and pYES2-ATMYB59 under osmotic stress. Yeast cells transformed with the pYES2 empty vector, pYES2-ATNAC3, pYES2-ERF107, pYES2-ANAC019 and pYES2-ATMYB59 were spotted on SC-Ura medium in 2 mL aliquots of 10-fold serially diluted (1, 10^− 1^, 10^− 2^, 10^− 3^, 10^− 4^, and 10^− 5^) cultures and were then incubated at 30 °C for 36 h

To date, numerous efforts have been implemented to improve plant tolerance against drought stress by engineering TFs, and some promising results have been obtained through the genetic transformation method. We heterologously overexpressed four selected DEGs annotated as ATNAC3 (F01.PB8282), ERF107 (F01.PB3413), ANAC019 (F01.PB9565), and ATMYB59 (F01.PB10800) in the yeast strain INVSc1 using the pYES2 vector to investigate their possible roles when yeast was exposed to 30% PEG and 5 M NaCl (Fig. 11). There was no difference in the survival rates between the pYES2 and four TF transgenic yeast cells under non-stress conditions. After being cultured in 30% PEG and 5 M NaCl for 36 h, all transformed lines survived well, but the control line was inhibited, especially under salt stress. These results agree with the expression pattern of homologous genes in *Arabidopsis*, which are also induced by dehydration stress in *Arabidopsis* [31–33]. Among the four TFs, the strain overexpressing ANAC019 survived better than the strains overexpressing other TFs under salt stress, indicating that ANAC019 proteins may confer dehydration tolerance to yeast cells. Because the current functional understanding of common vetch genes is limited, these results will provide candidate resources for subsequent studies of functional gene characterization in common vetch.

## Discussion

### High-quality transcripts were obtained from common vetch by PacBio Iso-Seq

Single-molecule sequencing offers longer read lengths and higher consensus accuracies, which enable the generation of full-length transcripts and near reference-quality genome assemblies, and the accuracy of genome annotation and transcriptome characterization are greatly enhanced, especially for species without reference genomes [23, 24]. In the present study, a total of 30,427 full-length transcripts were generated; which is much longer than the previously reported common vetch transcriptome data, such as 772 bp [25], 1124 bp [30] and 921.55 bp [34]. Of these full-length transcripts had a higher annotated percentage than that recognized in previous studies of common vetch (66.10 and 83.48%) [25, 30]. The remaining unannotated transcripts (528) may represent a common vetch-specific and novel gene pool, which will provide a good starting point in future experiments, including the preliminary functional characterization and investigation of their potential roles in drought stress responses in common vetch.

### Characterization of drought stress-related DEGs

Hybrid sequencing strategies have been established to make use of more accurate NGS short reads in conjunction with PacBio full-length transcripts, which can obtain higher-quality transcripts than the use of sequencing alone. Because the metabolic and biological processes of the above- and underground tissues of common vetch under drought stress are still unclear, we further investigated the expression patterns of DEGs in common vetch leaves and roots under drought conditions using the Illumina HiSeq X Ten platform. Compared with roots, more DEGs were identified in leaves. This observation indicated that in the roots, the expression profile of most genes was more stable than that in the leaves, but the number of DEGs detected in the roots was less than that in the leaves. A total of 799 DEGs were differentially expressed in both tissues, and only 57 DEGs showed an opposite expression profile in the two tissues, suggesting that the above- and underground tissue of common vetch may highly coordinate to optimize whole-plant adaption in drought stress by undergoing similar biological processes; additionally, distinct developmental trajectories still occurred in response to drought stress, which agrees with the results obtained from *Prunus mahaleb* and *Prunus persica* under drought stress [18, 20].

### Functional classification of transcriptional responses to drought in common vetch

After GO annotation, DEGs were labelled with 49 functional groups within the BP, MF, and CC categories. Furthermore, GO enrichment was conducted to identify the most dominant terms, and our results agreed with previous studies, indicating the conserved function between different plant species in response to drought stress [20, 35]. Markedly, “response to water” was the most enriched term in the leaves, with a total of 17 DEGs (all annotated as dehydrins) enriched, and all of them were upregulated; three of them were also upregulated in the roots. Dehydrins, also known as late embryogenesis abundant (LEA) proteins, are the best-studied group of LEA proteins [36]. qRT-PCR showed five *Prunus mume LEAs* upregulated in leaves under one or several treatments, especially under PEG treatment. When overexpressed in tobacco, four of these *LEAs* enhanced the tolerance of tobacco to cold and drought stresses [37]. Recently, many studies have shown that dehydrin genes and other protective mechanisms, such as the dismutation of reactive oxygen species (ROS), cooperatively improve plant tolerance to various stresses [38–40]. Among the co-induced DEGs, “proline biosynthetic process” was the most enriched term in the BP category. A total of nine P5CSs were enriched in the “proline biosynthetic process” term, and all of them were upregulated. As a key gene, *P5CS1*, involved in the biosynthesis of proline, has been proposed to play an important role under drought stress. In barley, thirteen unique haplotypes were identified from forty-one variations in *HvP5CS1*, and two haplotypes and five polymorphisms were significantly linked with drought tolerance-related traits [41]. The dehydrin and *P5CS* genes identified in this study might represent important candidate genes for further investigation of poorly understood drought-related genes in common vetch.

### Generic signalling pathways involved in the response of common vetch to drought stress

Under adverse situations, plants have evolved precise mechanisms that can benefit from distinguishing stress signals. Hormones play critical roles in plants during their adaption to adverse environmental conditions, such as auxins, ABA, CK, BR, ET, GA, JA, salicylic acid (SA) and strigolactones [42, 43]. Among them, ABA, ET, JA, and SA have been identified to play major roles in regulating plant responses to abiotic stresses [42, 44]. Under osmotic conditions, ABA is known to stimulate stomatal closure, maintaining water balance through the regulation of stress-responsive genes [45]. Thus, the ABA pathway can be considered the most important for common vetch drought tolerance, owing to the highly enriched DEGs in this study.

Previous studies indicated that PYR/PYL/RCAR had inhibitory activity similar to that of ABA negative regulators that inhibit the activity of Group A protein phosphatase type 2C (Group A *PP2C*s) in an ABA-dependent manner. For example, the Group A PP2Cs protein HAB1 functions as a negative regulator of ABA signalling in *Arabidopsis* [46, 47]. The ABA-receptor *PYL5* activates ABA signalling through the inhibition of Group A *PP2C*s and improved drought resistance in *Arabidopsis* [48]. A previous study evaluated the drought resistance of 14 transgenic *Arabidopsis*-overexpressing *PYLs* and showed that *PYL9* can promote drought resistance by inhibiting phosphatase activities [49]. A *PYL4 A194T* mutant uncovereds an important role of the *PYL4*/*PP2C*s interaction in ABA signalling, showing decreased stomatal conductance and improved water use in *Arabidopsis* [50]. A study of clade A *PP2C*s in the moss *Physcomitrella patens* found that Group A *PP2C* has a conserved effect on the regulation of the ABA response and drought resistance of land plants, indicating that Group A *PP2C* plays a key role in promoting the evolution of land plants [51, 52]. In addition, Group A *PP2C* genes identified in *Arabidopsis*, rice, maize, tomato, alfalfa, and cucumber were highly inducible in response to ABA signalling and abiotic stresses, indicating that Group A *PP2C* is highly conserved in land plants [53]. In our research, 17 (16 upregulated and one downregulated) and 11 (all upregulated) Group A *PP2C* DEGs were identified in the leaves and roots, respectively. Among them, seven and one PP2C (*ABI,* annotated as ABA Insensitive1) genes identified in the leaves and roots were all upregulated. Notably, the 11 *PP2C* DEGs identified in the roots were also differentially expressed in the leaves, and all of them were upregulated in both tissues, indicating that the ABA signalling process is highly coordinated to optimize whole-plant adaption in drought stress. In contrast, four and five *PYL* (annotated as PYRABACTIN RESISTANCE1/PYR1-LIKE) genes identified in the leaves and roots were all downregulated, two of which were co-induced in both tissues. The sucrose non-fermenting 1-related protein kinase (SnRKs) gene family plays vital roles in linking the stress response and metabolic responses under adverse conditions in plants; among these, the SnRK2 family is an osmotic-stress activated protein kinase [3]. Ten of nine *Arabidopsis* SnRK2s can be activated by osmotic stress; among these, SnRK2.7 and SnRK2.8 play important roles in regulating drought-responsive genes [54, 55]. The AREB/ABF-SnRK2 pathway functions in ABA/stress signalling via ABRE-mediated transcription that cooperatively regulates target genes associated with dehydration stress responses in plants [56]. In this study, a total of six and three common vetch SnRK DEGs were identified in the leaves and roots, two of which were downregulated in both tissues. One common vetch SnRK2 DEG was downregulated in both tissues, while two SnRK2 DEGs were specifically upregulated in the leaves and roots. These SnRKs may work as signal transducers and further function as essential controllers by phosphorylating stress-responsive genes in the whole common vetch plant under drought stress. The above results revealed that SnRK2s play important roles in ABA signal transduction pathways associated with ABA-PYL release and that the binding and inhibition of the PP2Cs to regulate downstream factors are significantly induced under drought stress in common vetch. This elaborate ABA-dependent response and the ability of these genes to respond to drought stress make them ideal candidates for further functional analysis in common vetch.

Starch and sucrose metabolism can maintain turgor pressure and leaf water content, depending on the genes encoding the enzymes involved in starch degradation, and can then change sucrose and amino acid contents under drought stress [35, 57, 58]. It is known that α/β-amylase and sucrose synthase activities are linked to the modification of plant carbon metabolism under drought conditions [59]. In our study, nine leaf-specific transcripts were annotated as “sucrose synthase”, and eight of them were upregulated. Three upregulated transcripts and one downregulated transcript encoding α-amylases were identified in the leaves, and five upregulated *α*-amylases were identified in the roots. Among these, three co-induced transcripts were upregulated in both tissues. Three and two common vetch transcripts were identified as *β*-amylase in the leaves and roots, respectively, belonging to glycosyl hydrolase family 14. More upregulated transcripts were identified in the leaves than in the roots. Our results indicated that drought resistance in common vetch is interconnected with carbon metabolism; the plant stabilizes the reserved energy from starch and glucose to mitigate drought stress.

**Fig. 12:**
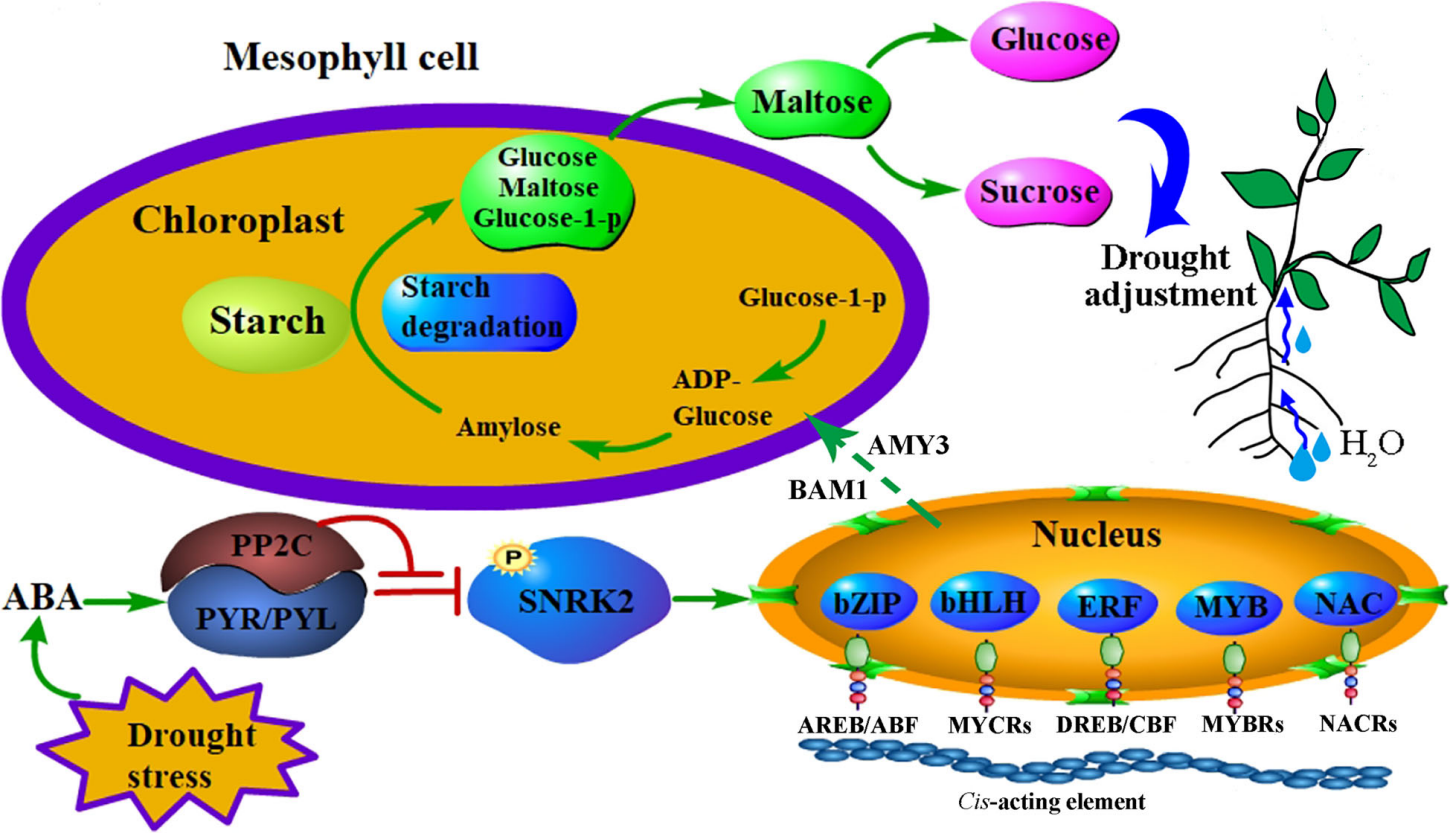
Proposed model of “plant hormone signal transduction” and “starch and sucrose metabolism” during drought stress. ABA controls the activity of BAM1 and AMY3 through the AREB/ABF-SnRK2 kinase-signaling pathway, and resulting a fraction of the maltose released from starch, and metabolized into sucrose and free hexoses. Then the sucrose exported to the root to enhance drought resistance. The solid and broken arrows indicate activation and speculative regulation, respectively, whereas lines ending with a bar show negative regulation

In *Arabidopsis,* the glucuronokinase *atglcak* mutants showed hypersensitivity to ABA, elevated water loss, reduced root development and impaired drought tolerance [60]. A previous study demonstrated that sugar accretion remained primarily owing to the increased hexose content through the decreased expression of the hexokinase gene, which enhanced the sucrose level upon the highest expression of the ABA-dependent AREB/ABF-SnRK2 signalling pathway [61]. Recent studies have shown that the regulation of starch in leaves triggered by *β*-AMYLASE1 (BAM1) and *α*-AMYLASE3 (AMY3) through the AREB/ABF-SnRK2 kinase-signalling pathway is important for osmotic stress tolerance and suggest that this mechanism is most likely conserved among different plant species [58, 62]. Here, we identified one common vetch *AMY3* gene that was upregulated in both tissues and two common vetch *BAM1* genes upregulated in the leaves. We speculated that ABA (AREB/ABF-SnRK2 pathway) regulates the activity of AMY3 and BAM1 to induce starch degradation in the leaves and increases carbon export to the roots, resulting in osmolyte accumulation and root growth for water and nutrient uptake during drought stress responses in common vetch (Fig. 12).

Compared with the “plant hormone signal transduction” and “starch and sucrose metabolism” pathways, “arginine and proline metabolism” is the third most important pathway enriched in common vetch leaves and roots. Some recent findings also showed that arginine, proline, and amines could regulate cellular osmotic adjustment, stabilize proteins and enzymes, and prevent cell membrane injury during water-limited conditions, indicating a positive association between amino acid metabolism, accumulation and turnover in drought tolerance in plants [18, 63–65]. In tobacco, the expression of three *PDH* genes (the key enzyme in proline degradation) was persistently inhibited both in leaves and roots from weak (1-day) to strong (6-d) dehydration conditions, resulting in 5–10 times higher proline concentrations in plants under dehydration conditions than the plants under control conditions [66]. In this study, *PDH*-annotated genes were specifically expressed in leaves, and all of them were downregulated, indicated a significant role of *PDH* genes in regulating the free proline content in common vetch. In plants, many *ALDH* family members respond to many abiotic stresses, for example wheat *traeALDH7B1-5A* genes enhanced drought resistance in transgenic *Arabidopsis*, maize *ALDH22A1* genes were induced by various abiotic stresses and transgenic plants overexpressing *ALDH22A1* improved various abiotic stress tolerance in tobacco [67, 68]. In common vetch, the amino acid kinase family and *ALDH* family genes were prominently enriched in both tissues, and all *ALDHs* were upregulated in the leaves. The pathway of arginine and proline metabolism strengthened after protein invention and/or with improved stress-induced protein collapse, as enlarged contents of specific amino acids underwent a marked increase in common vetch under drought conditions.

### The role of TFs in response to drought stress

TFs, as master regulators of many stress-responsive genes, are potential genomic candidates for enhancing tolerance to abiotic stresses such as drought because of their role in enabling plants to withstand unfavourable environments [12, 69]. It is now well established that several TF families, including bZIP (mainly AREB/ABF), AP2/ERF, NAC, bHLH, WRKY, and MYB, are key regulators that participate in various abiotic stresses [5, 70–72]. Among AREB/ABF subfamily genes in *Arabidopsis*, the expression of four AREB/ABF TFs (ABF1, ABF3, AREB1/ABF2 and AREB2/ABF4) was induced by abiotic stresses in vegetative tissues; except for ABF1, the remaining TFs were significantly induced both by osmotic and ABA stresses such as dehydration, and ABF2 is also likely involved in glucose signalling [56, 73, 74]. The overexpression of these TFs demonstrated that three AREB/ABFs function as master TFs of ABA signalling during drought stress in transgenic *Arabidopsis* plants [74, 75]. In this study, two common vetch transcripts were annotated as the “*ABF2*” gene in response to drought treatment, with one upregulated in both tissues and the other significantly upregulated only in the leaves, thus confirming their relevance in drought stress in common vetch. bHLH122 functions as a positive regulator of osmotic stress resistance in *Arabidopsis* and increases cellular ABA levels by repressing CYP707A3 transcripts [76]. Three common vetch transcripts were annotated as “bHLH122”, and among them “F01. PB32082” was upregulated in both tissues, while “F01. PB9425” and “F01. PB13415” were upregulated and downregulated in leaves, respectively. Transcript “F01. PB6002”, annotated as “*MYB48*”, was upregulated in both tissues after drought treatment. Previously, the overexpression of a maize *ZmMYB48* gene markedly improved drought resistance in transgenic *Arabidopsis* plants; moreover, the expression level of stress/ABA-responsive genes and the ABA content also increased under drought stress [77]. In this study, a total of 18 and 5 WRKY transcripts were identified in the roots and leaves, respectively, and all of the transcripts identified in the roots were repressed. A previous study showed that mild osmotic conditions quickly prevent the growth of aboveground shoots, whereas roots continue to elongate [78]. In addition, when the expression of the *WRKY75* gene was suppressed, the lateral roots and root hairs were significantly increased, which negatively regulates root growth [79]. The WRKY transcripts downregulated in the roots may influence lateral root and root hair formation and increase the total surface area of roots, and these changes can improve the ability of common vetch to grow in soils where water is limited. However, more common vetch ERF transcripts were expressed in the leaves than in the roots; four of them were significantly expressed in both tissues, while nine and three of them were specifically expressed in the leaves and roots, respectively. Recent studies have indicated that the ERF protein family can improve drought tolerance in a variety of plant species. *OsERF48* acts as a positive regulator that contributes to root growth and drought resistance in rice [80]. DREB1/CBF-type proteins belong to the (AP2/ERF)-type TF family and participate in the regulation of drought, heat and cold stress-responsive gene expression in soybean and *Arabidopsis* [81]. DREB1A could improve drought stress tolerance by regulating gas exchange and production traits in soybean [82]. The number of up- and downregulated genes among the four co-response *ERF* genes in common vetch was equal, and one transcript annotated as DREB1F (F01. PB1045) was upregulated in both tissues. Notably, nearly 70% of *ERF* family members were upregulated in the leaves, suggesting that these *ERF* genes may be more sensitive to drought in common vetch leaves. Moreover, the zinc-finger transcription factor family is another important TF that plays important functions in the response of plants to drought stress [83–85]. The overexpression of *OsC3H47* promotes drought tolerance and decreases ABA sensitivity in rice [85]. Tobacco plants overexpressing *TaNF-YB3;l* exhibited improved drought tolerance through the modulation of the ABA-associated signalling pathway [86]. Most of these TF family members exhibited an inducible expression profile after drought stress in common vetch leaves and roots. Taken together, diverse expression patterns of specific or co-responsive TFs showed their involvement in the drought response in an ABA-dependent manner in the two common vetch tissues, indicating that TF family members play a vital role in modulating leaf and root signal transduction and may participate in crosstalk at many steps.

## Conclusions

In this study, for the first time, we comprehensively presented and comparatively analysed the global transcriptional regulation of common vetch leaves and roots under drought stress. These sequences were assembled into 30,427 full-length transcripts, with an average length of 2278.89 bp. A total of 3464 and 3062 DEGs were identified in the leaves and roots, respectively. Crosstalk and divergence among DEGs were identified in the cascades of the molecular networks between the leaf and root tissues under drought stress. Hormone signal transduction, starch and sucrose metabolism, and arginine and proline metabolism were extensively enriched pathways. Genes involved in the AREB/ABF-SnRK2 pathway may regulate the activity of AMY3 and BAM1 to induce starch degradation in the leaves and increase carbon export to the roots to enhance drought tolerance. Furthermore, heterologous expression experiments in yeast revealed that four TFs can act as candidate genes to improve osmotic stress tolerance. Overall, the inventory of drought-responsive transcripts in leaves and roots increases our understanding of the above- and underground biological characteristics of common vetch under water-limited conditions.

## Methods

### Stress treatments and sample collection

Healthy seeds of the common vetch cultivar “Lanjian No.1” were provided by Lanzhou University (Lanzhou, Gansu, China). Firstly, seeds were surface sterilized in 1.0% (v/v) sodium hypochlorite for five mins, washed six times with distilled water, and then allowed to germinate for 4 days at 20 °C. Thereafter, 20 uniformly germinated seeds were separately sown in 60-well plates and hydroponically cultured with 1/2 MS (half-strength Murashige and Skoog) solution (pH = 5.8). The seedlings were subsequently moved to a greenhouse at 20 °C, 16 h/8 h (light/dark), 180 μmol m^− 2^ s^− 1^ photosynthetically active radiation and 80% humidity, and the 1/2 MS solution was changed every 2 days. To optimize the PEG concentration, four-day-old seedlings were transplanted into 1/2 MS solution containing different concentrations of PEG (0, 10, 15, 20, 25, 30 and 35%) for 7 days. Then, the above- and underground length and fresh weight of the seedlings were measured. Five biological replicates were performed.

Seedlings were grown for 7 days in 1/2 MS solution after being sown, and ten healthy seedlings at the four-leaf stage with uniform and strong growth were selected and equally classified into two groups: one was transferred into control pots, and the other group was transferred into pots containing a solution of equal parts of PEG (20% (m/V), pH = 5.8) and half-strength medium solution for drought treatment. To reduce the circadian rhythm effects, the leaves and roots of each sample from the control and drought treatment were grown in parallel, harvested after 24 h, immediately frozen in liquid nitrogen and stored at − 80 °C. Each sample was from four different seedlings, and three biological replicates were collected at each time point.

### RNA isolation and assessment

A total of 12 samples [2 tissues (leaf and root) × 2 treatments (control and 20% PEG/24 h) × 3 biological replications] were used for transcriptome analysis. Total RNA was extracted using the RNeasy Plus Mini Kit (Agilent Technologies, CA, USA). Subsequently, RNA contamination and degradation were assessed on 1% agarose gels. The RNA integrity was assessed using the RNA Nano 6000 Assay Kit and an Agilent 2100 Bioanalyzer. For PacBio isoform sequencing (Iso-Seq) (Pacific Bioscience, Menlo Park, USA), equal amounts of total RNA with an RNA integrity number ≥ 7.0 and a 28S/18S ratio ≥ 1.0 from each sample pooling were carried out. For Illumina sequencing (Illumina, San Diego, USA), an indexed library of 12 internodal RNA samples was prepared and sequenced using the Illumina HiSeq X Ten platform.

### PacBio Iso-Seq library construction, sequencing, and data analysis

The sequencing library was prepared according to the official protocol with the following modifications. The SMARTer™ PCR cDNA Synthesis Kit (Clontech, CA, USA) was used to synthesize full-length cDNA from 4 μg of mixed total RNA. After PCR amplification, the BluePippin Size Selection System (Sage Science, Beverly, MA, USA) was used to select product sizes, and then three libraries were produced, corresponding to fragments of 1–2, 2–3 and 3–6 kb for each sample length. The amplified cDNA products were then subjected to the construction of SMRTbell Template libraries according to the Iso-Seq protocol [87, 88]. Further, Qubit2.0 and Agilent 2100 were used to confirm accurate quantification and library size, respectively; only when the library size met the expected criteria was sequencing performed. Finally, a total of 7 SMRT cells were sequenced on the PacBio RSII platform.

The raw data were processed into error-corrected reads of insert (ROIs) using the ToFu pipeline with default parameters. Next, non-full-length (nFL) and full-length non-chimeric (FL) transcripts were determined by searching for the polyA tail signal and the 5′ and 3′ cDNA primers in the ROIs. The Iterative Clustering for Error Correction (ICE) method was used to obtain consensus isoforms, and FL consensus sequences from ICE were polished using Quiver. Further, polished consensus reads were acquired from the original consensus reads corrected with nFL reads, and FL transcripts with post-correction accuracy above 99% were generated for further studies. High-quality Iso-Seq FL transcripts were used, and redundancy was removed using CH-HIT (identity > 0.99) to obtain the transcripts.

### Illumina transcriptome library preparation, sequencing, and data analysis

After treatment with DNase I (TaKaRa, Dalian, China), mRNA was purified from total RNA with magnetic oligo beads. The sequencing libraries were generated using the NEBNext UltraTM RNA Library Prep Kit for Illumina (NEB, USA) according to the manufacturer’s protocols. The cDNA library was sequenced with a 100-bp paired-end format using the Illumina HiSeq X Ten, and each sample yielded more than 6 Gb of clean data.

Raw data in the fastq format were first processed using internal Perl scripts. In this step, clean data were obtained by removing three kinds of reads, which were reads containing adaptors, low-quality reads and reads with more than 10% unknown bases. Meanwhile, the parameters of Q30 and the GC content were used to estimate the quality of these clean data.

### Comparison with full-length transcripts and quantification of gene expression levels

The isoform sequences were corrected with the NGS data using the Long-Read de Bruijn Graph Error Correction (LoRDEC) tool (−t 5 -b 200 -e 0.4 -s 3 k-mers 21 and 25). Then, the clean data were mapped back onto the assembled transcriptome database using Bowtie2, and the read count for each transcript was acquired from the mapping results. The transcript expression levels were identified by the RSEM software package and calculated by the fragments per kilobase of transcript per million mapped transcript (FPKM) method for each sample [89]. Differentially expressed genes (DEGs) were determined by setting the thresholds for false discovery rate (FDR) < 0.01 and the log_2_(Group1/Group2) ≥ 1 by performing pairwise comparisons for the treatment and control samples.

### Functional annotation of transcripts and DEG analysis

Transcripts were annotated by performing BLASTX searches against public databases, including the National Center for Biotechnology Information (NCBI)non-redundant protein database (Nr), Protein Family (Pfam), Clusters of Orthologous Groups of proteins (KOG/COG/eggNOG), Swiss-Prot, GO, and KEGG. DEGs were employed for the GO and KEGG pathway enrichment analysis using the GOseq R package and KOBAS (*P*-value< 0.05), respectively [90, 91]. DEGs were used to identify potential TFs from the PlantTFDB database (http://planttfdb.cbi.pku.edu.cn/).

### qRT-PCR analysis

The qRT-PCR was conducted using a CFX96 Touch™ Real-Time PCR Detection System (Bio-Rad, USA) with SYBR® Green and analysed with CFX Manager software (Bio-Rad). The PCR was programmed as follows: 95 °C for 3 min and 39 cycles of 95 °C for 10 s and 55 °C for 30 s. The specific primers were designed by using Primer3 as shown in Additional file 6: Table S6, and their specificity was confirmed by a BLAST search of the common vetch transcripts. As an internal standard, the common vetch actin gene (*Unigene 68,614*) was selected to calculate the relative fold expression levels according to the *Ct* method.

### Expression vector construction and stress tolerance tests of transgenic yeast

The complete coding sequence of four TFs were isolated from common vetch leaves and roots with PCR primer pairs as shown in Additional file 7: Table S7. Then, the PCR products of those TFs were inserted into the yeast expression pYES2 vector (Invitrogen, Carlsbad, USA). Subsequently, the expression vector and an empty pYES2 control plasmid were introduced into the INVSc1 yeast strain (Invitrogen, USA) through the lithium acetate method [92]. The transformants were then selected on SC medium devoid of uracil with 2% (w/v) glucose at 30 °C for 36 h. The osmotic tolerance evaluation was performed according to the methods described previously [93, 94].

## Supplementary information

**Additional file 1 Table S1.** The expression profile of novel transcripts.

**Additional file 2 Table S2.** The expression profile of oxidation-reduction process category enriched transcripts and their annotation in Pfam, Swissprot and nr database.

**Additional file 3 Table S3.** The expression profile of defense response category enriched transcripts and their annotation in Pfam, Swissprot and nr database.

**Additional file 4 Table S4.** KEGG enrichment results in common vetch leaves.

**Additional file 5 Table S5.** DEGs involved in the “Plant hormone signal transduction”, “Starch and sucrose metabolism” and “Arginine and proline metabolism” pathways under drought stress (Table S1a-c).

**Additional file 6 Table S6.** The qRT-PCR primers used in this study.

**Additional file 7 Table S7.** Primers used for yeast assays in this study.

**Additional file 8 Figure S1.** Investigation of the characteristics of PEG stress resistance in common vetch seedlings. Common vetch aboveground length (A), underground length (B) and fresh weight (C) under various concentrations of PEG (0, 10, 15, 20, 25, 30 and 35%) for 7 days.

**Additional file 9 Figure S2.** Scatter diagram indicating the expression changes of all detected transcripts (A), and novel transcripts (B) under drought stress in both tissues.

**Additional file 10 Figure S3.** The expression pattern of ten selected genes identified by RNA-Seq was verified by qRT-PCR in leaves and roots in the control and drought-stressed plants. The grey bars represent the relative expression determined by RT-qPCR (left y-axis) and the orange lines represent the level of expression (FPKM) of the transcripts (right y-axis).

**Additional file 11 Figure S4.** Validation of the expression (log_2_-fold change) of selected genes based on RNA-Seq via qRT-PCR. The results are plotted for genes that show up- or down-regulation in common vetch upon drought stress. The linear trend line and the *R*^*2*^-value are shown.

**Additional file 12 Figure S5.** The expression profile of the DEGs enriched in “response to water” (A), “response to stress” (B) and “proline biosynthetic process” (C) among leaves, roots and the DEGs shared between two tissues, respectively.

## Data Availability

The sequencing raw data for 12 samples produced by Illumina HiSeq X Ten can be accessed in the NCBI Sequence Read Archive (SRA) database with the link of https://www.ncbi.nlm.nih.gov/Traces/study/?acc=PRJNA554306, under accession number SRR9674916-SRR9674930, and the bioProject accession is PRJNA554306.

## Abbreviations

DEGs: Differentially expressed genes
TFs: Transcription factors
ABA: Abscisic acid
NGS: Next-generation RNA sequencing
PacBio: Pacific Biosciences
Nfl: Non-full-length
FL: Full-length non-chimeric
ICE: Iterative Clustering for Error Correction
LoRDEC: Long-Read de Bruijn Graph Error Correction
FPKM: Fragments per kilobase of transcript per million mapped transcript
FDR: false discovery rate
BP: The biological process
CC: The cellular component
MF: The molecular function
KEGG: The Kyoto Encyclopedia of Genes and Genomes
GO: Gene Ontology
CK: Cytokinin
BR: Brassinosteroid
ET: Ethylene
GA: Gibberellin
JA: Jasmonic acid
ALDH: Aldehyde dehydrogenase
PDH: Proline dehydrogenase
ROS: Reactive oxygen species
Group A *PP2C*s: Group A protein phosphatase type 2C
SnRKs: The sucrose non-fermenting 1-related protein kinase
