# Supplementary figures and images for: Coordinated mechanisms of leaves and roots in response to drought stress underlying full-length transcriptome profiling in Vicia sativa L
Source: BMC Plant Biol. 2020 Apr 15;20:165. doi: 10.1186/s12870-020-02358-8 (PMC7161134; doi:10.1186/s12870-020-02358-8)

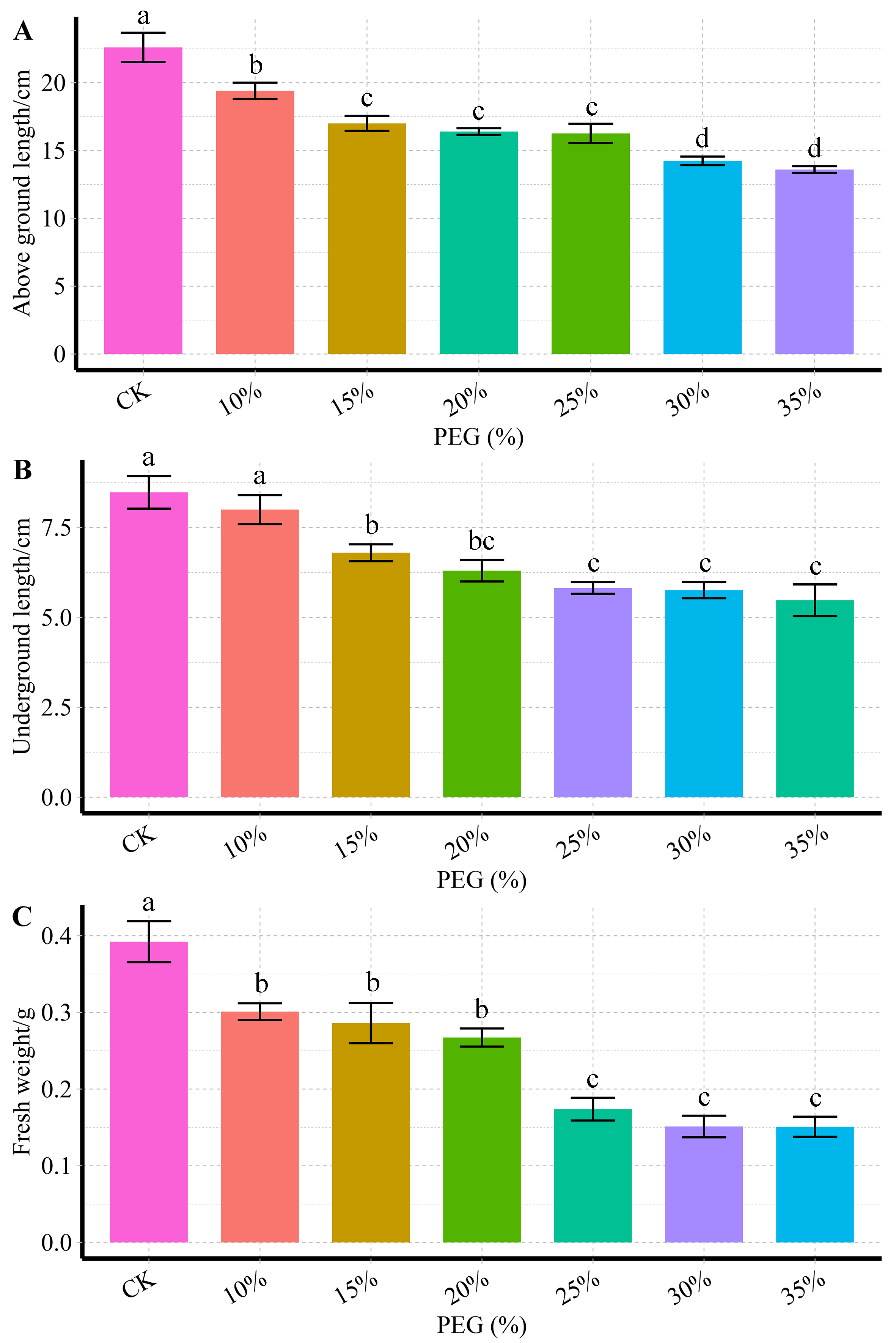

Supplement: Supplementary file 8 — Additional file 8 Figure S1. Investigation of the characteristics of PEG stress resistance in common vetch seedlings. Common vetch aboveground length (A), underground length (B) and fresh weight (C) under various concentrations of PEG (0, 10, 15, 20, 25, 30 and 35%) for 7 days. [file 12870_2020_2358_MOESM8_ESM.tif]

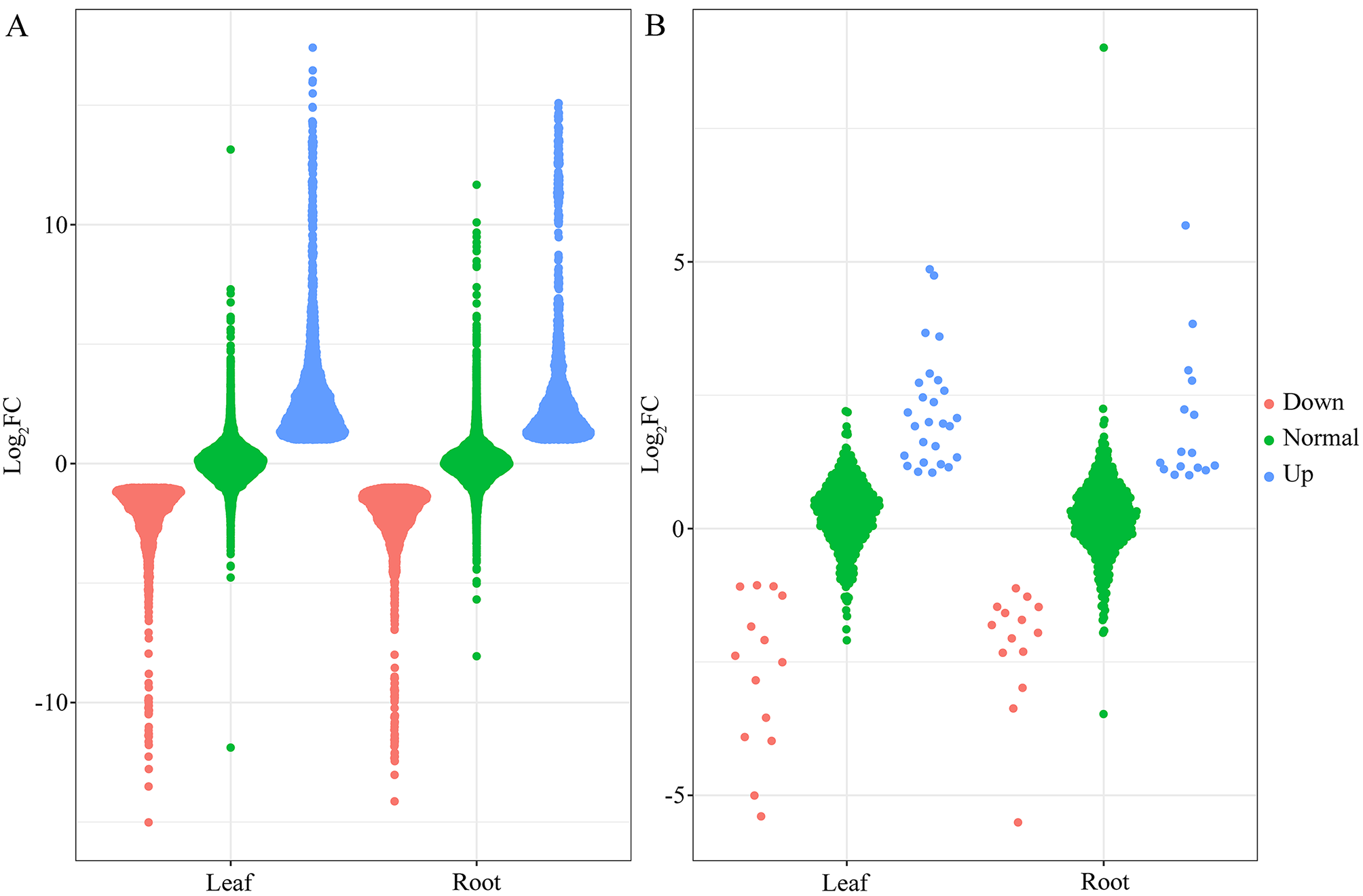

Supplement: Supplementary file 9 — Additional file 9 Figure S2. Scatter diagram indicating the expression changes of all detected transcripts (A), and novel transcripts (B) under drought stress in both tissues. [file 12870_2020_2358_MOESM9_ESM.tif]

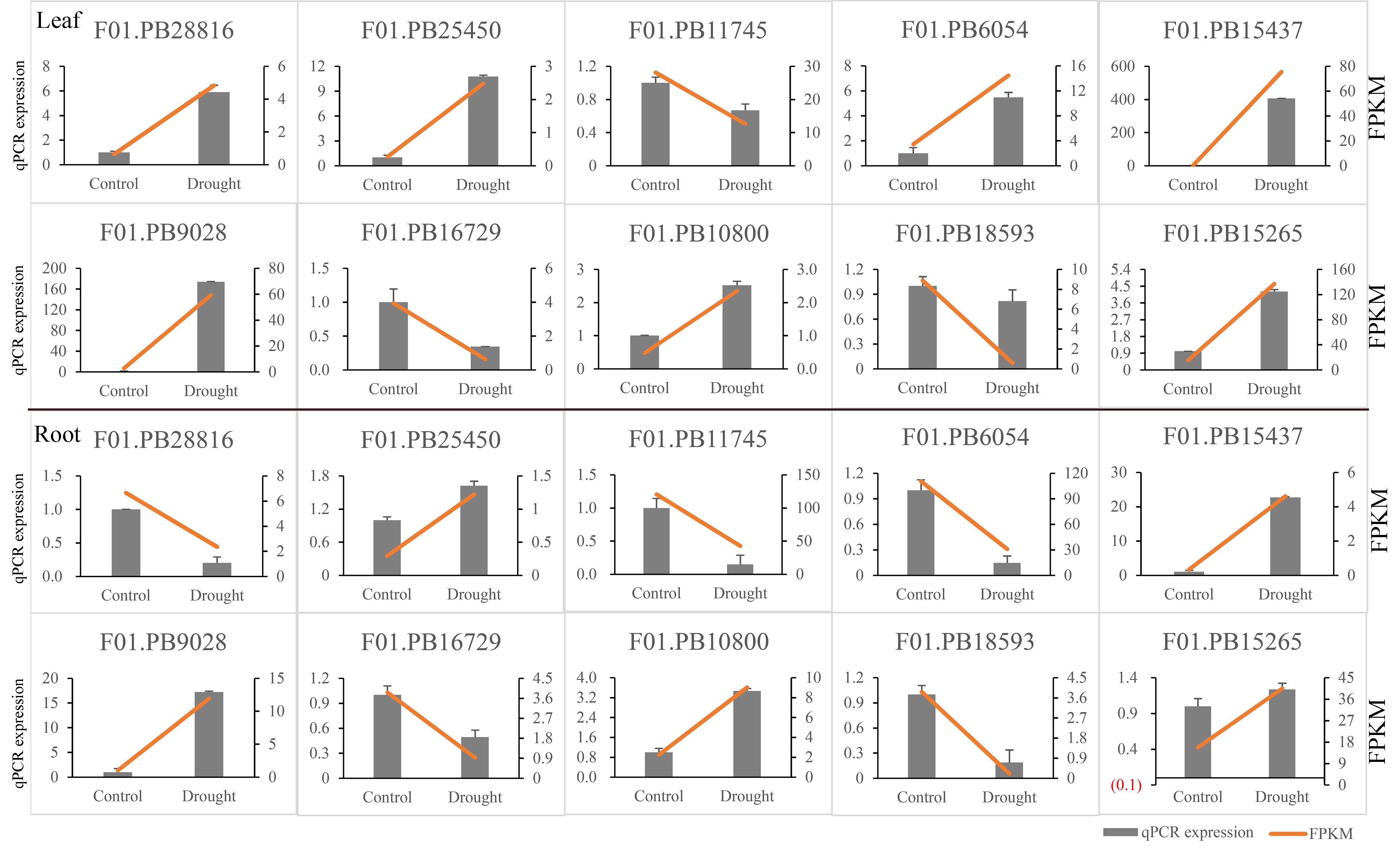

Supplement: Supplementary file 10 — Additional file 10 Figure S3. The expression pattern of ten selected genes identified by RNA-Seq was verified by qRT-PCR in leaves and roots in the control and drought-stressed plants. The grey bars represent the relative expression determined by RT-qPCR (left y-axis) and the orange lines represent the level of expression (FPKM) of the transcripts (right y-axis). [file 12870_2020_2358_MOESM10_ESM.tif]

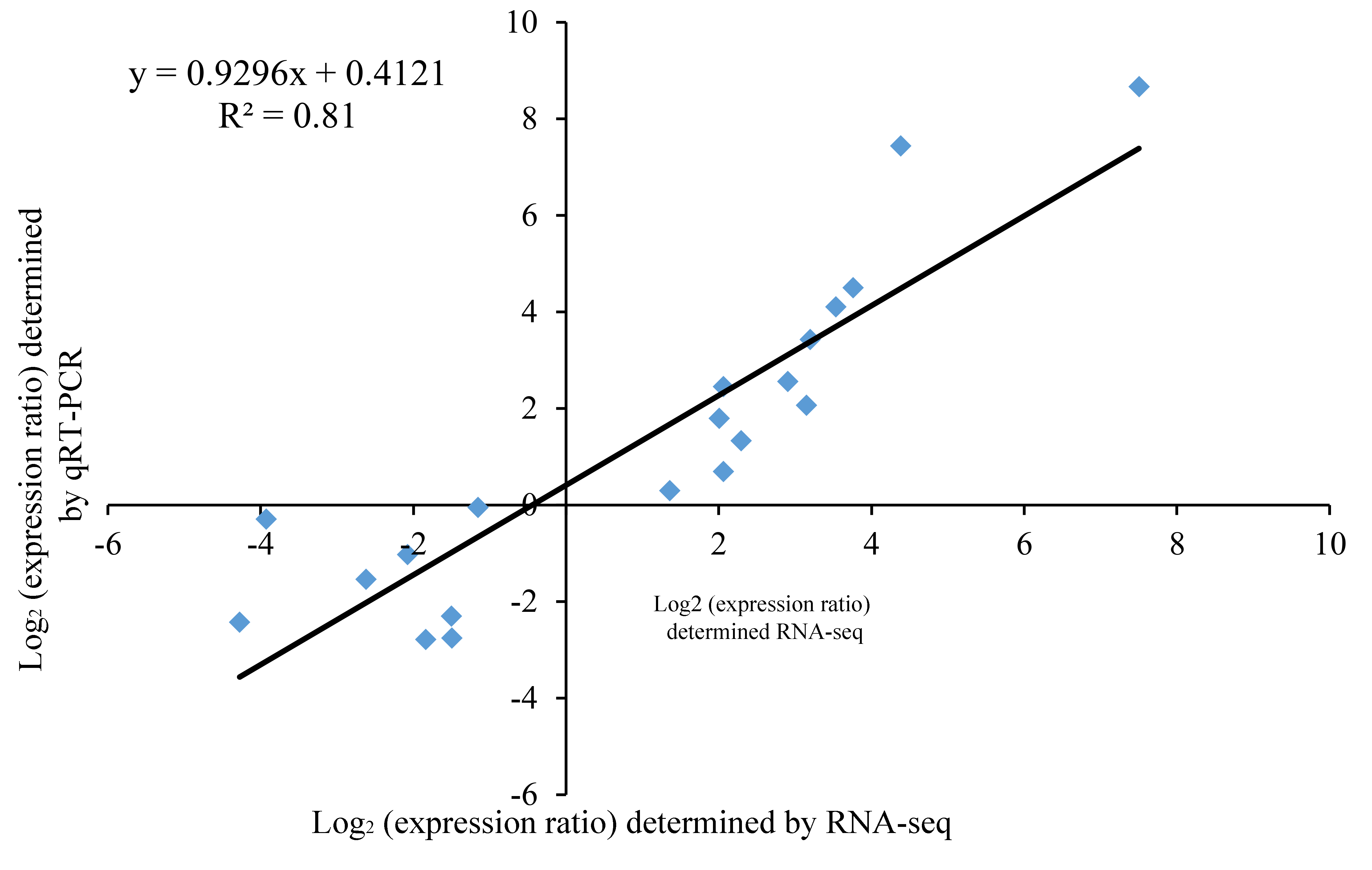

Supplement: Supplementary file 11 — Additional file 11 Figure S4. Validation of the expression (log2-fold change) of selected genes based on RNA-Seq via qRT-PCR. The results are plotted for genes that show up- or down-regulation in common vetch upon drought stress. The linear trend line and the R2-value are shown. [file 12870_2020_2358_MOESM11_ESM.tif]

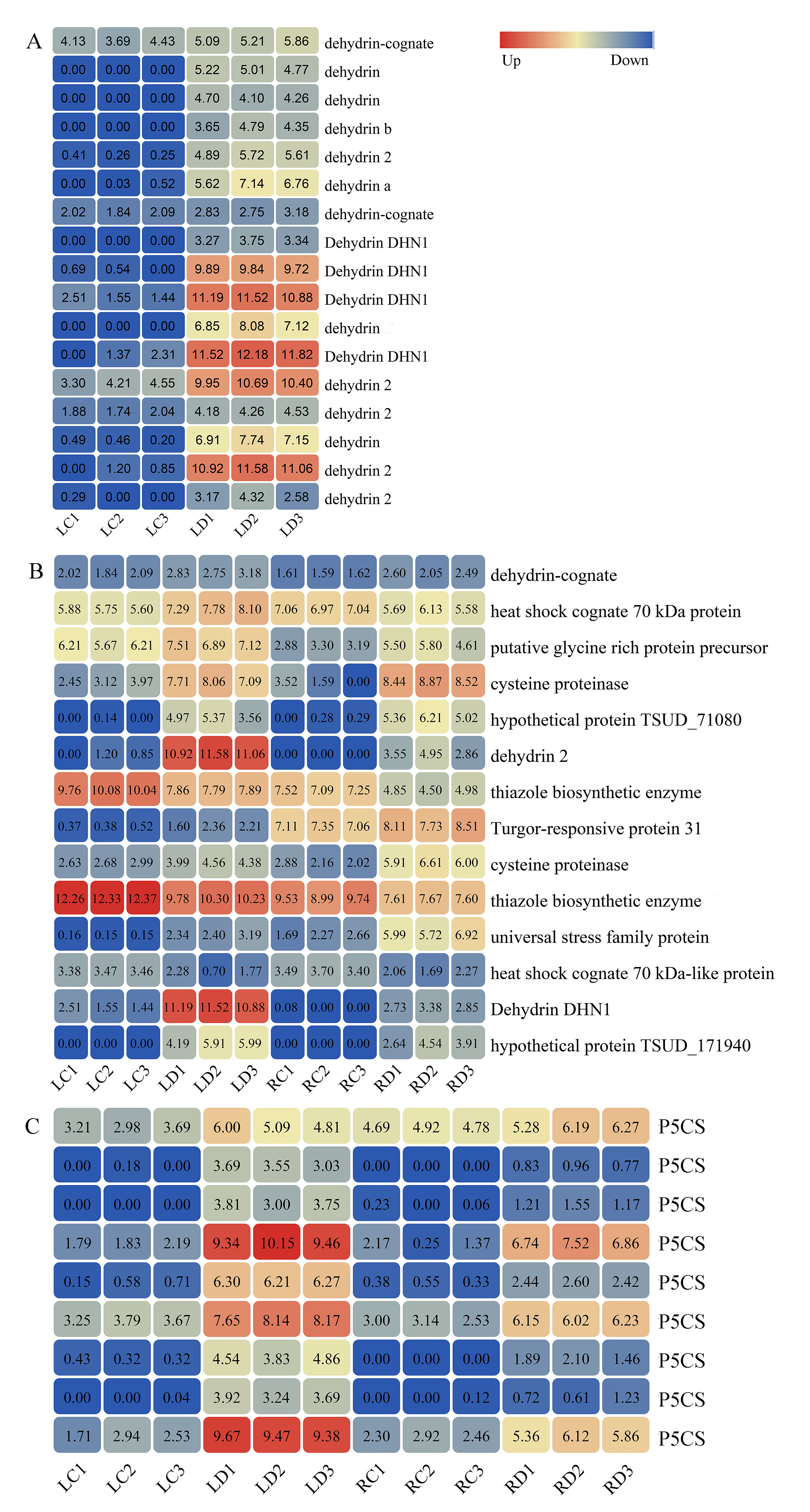

Supplement: Supplementary file 12 — Additional file 12 Figure S5. The expression profile of the DEGs enriched in “response to water” (A), “response to stress” (B) and “proline biosynthetic process” (C) among leaves, roots and the DEGs shared between two tissues, respectively. [file 12870_2020_2358_MOESM12_ESM.tif]
